# Dipeptidyl Peptidase 4/Midline‐1 Axis Promotes T Lymphocyte Motility in Atherosclerosis

**DOI:** 10.1002/advs.202204194

**Published:** 2023-01-22

**Authors:** Xiaoquan Rao, Michael Razavi, Georgeta Mihai, Yingying Wei, Zachary Braunstein, Matthew B. Frieman, Xiao Jian Sun, Quan Gong, Jun Chen, Gang Zhao, Zheng Liu, Michael J. Quon, Lingli Dong, Sanjay Rajagopalan, Jixin Zhong

**Affiliations:** ^1^ Division of Cardiology Department of Internal Medicine Tongji Hospital Tongji Medical College Huazhong University of Science and Technology Wuhan Hubei 430030 P. R. China; ^2^ Cardiovascular Research Institute Case Western Reserve University Cleveland Ohio 44106 USA; ^3^ Brigham and Women's Hospital Harvard Medical School Boston Massachusetts 02115 USA; ^4^ Wexner Medical Center The Ohio State University Columbus Ohio 43210 USA; ^5^ Department of Microbiology and Immunology University of Maryland School of Medicine Baltimore Maryland 21201 USA; ^6^ Department of Medicine University of Maryland School of Medicine Baltimore Maryland 21201 USA; ^7^ Department of Immunology School of Medicine Yangtze University Jingzhou Hubei 434023 P. R. China; ^8^ Sinopharm Dongfeng General Hospital Hubei University of Medicine Shiyan Hubei 442008 P. R. China; ^9^ Department of Cardiology Shandong Provincial Hospital affiliated to Shandong University Jinan Shandong 250021 P. R. China; ^10^ Department of Otolaryngology‐Head and Neck Surgery Tongji Hospital Tongji Medical College Huazhong University of Science and Technology Wuhan Hubei 430030 P. R. China; ^11^ Institute of Allergy and Clinical Immunology Tongji Hospital Tongji Medical College Huazhong University of Science and Technology Wuhan Hubei 430030 P. R. China; ^12^ Division of Rheumatology and Immunology Department of Internal Medicine Tongji Hospital Tongji Medical College Huazhong University of Science and Technology Wuhan Hubei 430030 P. R. China

**Keywords:** atherosclerosis, dipeptidyl peptidase‐4, midline‐1, migration, T cell

## Abstract

T cells play a crucial role in atherosclerosis, with its infiltration preceding the formation of atheroma. However, how T‐cell infiltration is regulated in atherosclerosis remains largely unknown. Here, this work demonstrates that dipeptidyl peptidase‐4 (DPP4) is a novel regulator of T‐cell motility in atherosclerosis. Single‐cell ribonucleic acid (RNA) sequencing and flow cytometry show that CD4^+^ T cells in atherosclerotic patients display a marked increase of DPP4. Lack of DPP4 in hematopoietic cells or T cells reduces T‐cell infiltration and atherosclerotic plaque volume in atherosclerosis mouse models. Mechanistically, DPP4 deficiency reduces T‐cell motility by suppressing the expression of microtubule associated protein midline‐1 (Mid1) in T cells. Deletion of either DPP4 or Mid1 inhibits chemokine‐induced shape change and motility, while restitution of Mid1 in *Dpp4^−/−^* T cell largely restores its migratory ability. Thus, DPP4/Mid1, as a novel regulator of T‐cell motility, may be a potential inflammatory target in atherosclerosis.

## Graphical abstract

A novel mechanism regulating T‐cell infiltration into atherosclerotic plaque is reported here. Dipeptidyl peptidase 4 (DPP4) is identified as a crucial molecule promoting T‐cell migration in atherosclerosis via midline‐1‐dependent cytoskeletal rearrangement. The nonenzymatic function of DPP4 may serve as a potential therapeutic target for atherosclerotic disease.

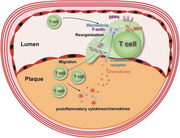

## 1 Introduction

T lymphocytes, as a key cellular component of atherosclerotic plaque, play an important pathogenic role in the development of atherosclerosis.^[^ ^1^ ^]^ T cells infiltrate into the vascular wall, chronologically preceding the formation of atheroma.^[^ ^1b^ ^]^ However, how T‐cell infiltration into the atherosclerotic plaque is regulated in atherosclerosis remains largely unknown.^[^ ^2^ ^]^

Dipeptidyl peptidase‐4 (DPP4), also known as Cluster of Differentiation 26, is an exopeptidase that cleaves *N*‐terminal dipeptides from substrates including neuropeptides, chemokines, and incretin peptides.^[^ ^3^ ^]^ Its role in the cleavage of incretin hormones such as glucagon‐like peptide‐1 (GLP‐1), ‐2 (GLP‐2), and gastric inhibitory polypeptide (GIP) has however received the most attention. The inhibition of the catalytic function of DPP4 using small molecule pharmacologic agents has been shown to result in improvement in glycemic control, via increasing GLP‐1 and GIP levels, with low risk for side effects such as weight gain and hypoglycemia. However, the lack of coordinate reduction in major atherosclerotic events in humans has been disappointing and has lacked a credible mechanistic explanation.^[^ ^4^ ^]^ While GLP‐1 receptor agonists have been shown to reduce adverse cardiovascular events and cardiovascular mortality,^[^ ^5^ ^]^ the inhibition of DPP4 in cardiovascular diseases failed to yield a consistent result in human and animal studies. Animal studies have shown improved cardiac performance and reduced infarct size in DPP4 whole‐body knockout mice after ischemia‐reperfusion injury.^[^ ^6^ ^]^ In contrast, several large‐scale clinical trials in patients with type 2 diabetes showed no significant improvements in primary cardiovascular endpoints in patients treated with DPP4 enzymatic inhibitors compared to those with placebo.^[^ ^4^ ^]^ In addition, the GLP‐1 receptor antagonist exendin (9‐39) failed to abrogate the reduction of infarct size in DPP4 knockout rats.^[^ ^6^ ^]^ These results suggest that there may be GLP‐1‐independent mechanisms involved in the cardioprotection of DPP4 deficiency.

We and others have shown that the noncatalytic activity of membrane‐bound DPP4 could enhance T‐cell and dendritic cell‐mediated inflammation via interacting with ligands such as adenosine deaminase (ADA) and caveolin‐1.^[^ ^7^ ^]^ The catalytic function of DPP4 has also been linked to immune regulation via enzymatic cleavage of proinflammatory chemokines.^[^ ^3^, ^8^ ^]^ Therefore, the role of DPP4 in immune regulation may derive from both catalytic and noncatalytic activities and needs to be explored in detail in the pathogenesis of inflammatory diseases. In particular, given the obligatory role of T cells in atherosclerosis and the high levels of DPP4 expression in T cells, the role of T‐cell derived DPP4 in vascular inflammation and atherosclerosis deserves investigation in detail. In this study, we demonstrated a critical role for DPP4 in both human and murine atherosclerosis. We also discovered a previously unrecognized pathway regulating T‐cell motility, which may provide a mechanistic basis for the role of DPP4 in atherosclerosis and raise the possibility of targeting the noncatalytic function of DPP4 as a therapeutic strategy.

## 2 Results

### 2.1 CD4^+^ T Cells from Patients with Atherosclerosis Display a Unique Transcription Profile

**Figure 1:**
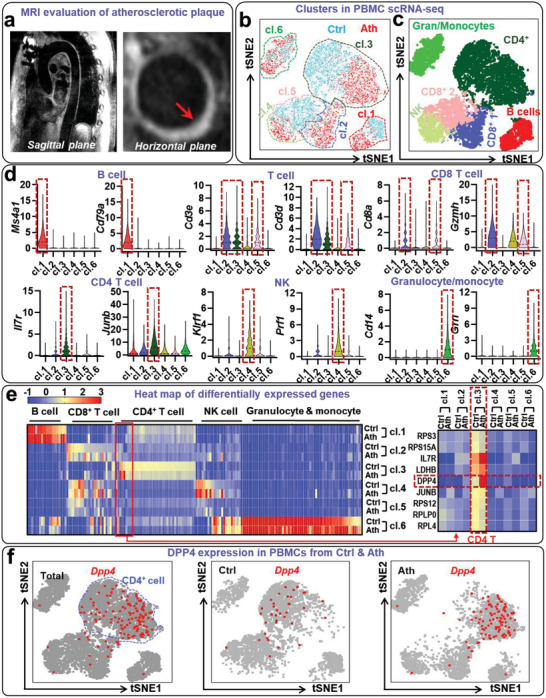
Single cell RNA profiling of circulating immune cells in patients with atherosclerosis and healthy controls: peripheral blood mononuclear cells (PBMCs) from four patients with atherosclerosis (Ath) and four healthy controls (Ctrl) were used for 10× single cell RNA sequencing. After quality control analysis, a total of 5151 cells from atherosclerosis patients and 5044 cells from control subjects were acquired. a) The presence of aortic atherosclerosis was confirmed by 3D MRI scan. b) tSNE plot showing distribution and clustering of cells from healthy controls (blue) or atherosclerotic patients (red). c) tSNE plot showing different PBMC clusters and their corresponding immune cell types. d) Violin plots showing representative marker genes of B cell (*Ms4a1*, *Cd79a*), T cell (*Cd3e*, *Cd3d*), CD8^+^ T cell (*Cd8a*, *Gzmh*), CD4^+^ T cell (*Il7r*, *Junb*), natural killer cell (*Klrf1*, *Prf1*), monocyte (*Cd14*), and granulocyte (*Grn*) in different clusters. e) Heat map showing top differentially expressed genes in each cluster from atherosclerosis and control group. f) tSNE plot showing dipeptidyl peptidase‐4 (DPP4) expression in cells from healthy controls or atherosclerotic patients. Left, DPP4 expression in all populations from both patients with atherosclerosis and healthy controls; middle, DPP4 expression in cells from healthy controls; right, DPP4 expression in cells from patients with atherosclerosis. Cells expressing DPP4 were shown in red color. *N* = 4 per group for figures b–f.

To characterize the immune transcriptome profile in human atherosclerosis, we performed single‐cell ribonucleic acid (RNA) sequencing on peripheral blood mononuclear cells (PBMCs) isolated from patients with known atherosclerosis and healthy control subjects. The presence of thoracic aortic atherosclerosis was confirmed by 3D magnetic resonance imaging (MRI) scanning (**Figure** **1**a). After removal of low‐quality cells, a total of 5151 PBMCs from four patients with atherosclerotic disease and 5044 PBMCs from four control subjects were analyzed. Unsupervised clustering showed that major immune populations were well separated in the tSNE plot (Figure 1b,c). We identified six distinct clusters representing major immune subpopulations: B lymphocytes (cluster 1, cl.1) characterized by *Ms4a1* (CD20) and *Cd79a* expression; CD8^+^ T lymphocytes (cl.2 and cl.5) characterized by expression of *Cd3d*, *Cd3e*, *Cd8a*, and *Gzmh*; CD4^+^ T lymphocytes (cl.3) characterized by expressions of *Cd3d*, *Cd3e*, *Il7r*, and *Junb*; natural killer cells (cl.4) characterized by *Klrf1* (killer cell lectin like receptor F1) and *Prf1* (perforin 1) expression; mixture (cl.6) of monocytes characterized by *Cd14* expression and granulocytes characterized by *Grn* (granulin). Each cluster was then separated into control (Ctrl) and atherosclerosis (Ath) groups based on the origin of the cells. Although controls and atherosclerotic patients showed a grossly similar transcriptomic signature in the same cluster, a distinctive expression pattern was noted for several genes in the two groups (Figure 1d,e). We found that *Dpp4* was mainly expressed in cl.3 (CD4^+^ T lymphocytes) and was further increased in CD4^+^ T lymphocytes from patients with atherosclerosis (Figure 1f and Figure S1, Supporting Information). In addition to *Dpp4*, there was an upregulation of a number of inflammation‐related genes including *Card8*, *Card16*, *Gzma*, *Prf1*, *Il2rg*, *Il32*, *Cd52*, and *Ccl4* in CD4^+^ T cells isolated from patients with atherosclerosis (Figure S1, Supporting Information). The predominant expression of *Dpp4* in T cells and upregulation of *Dpp4* in aorta‐infiltrating CD4^+^ T lymphocytes from patients with cardiovascular disease were also noted in the single cell RNA sequencing data^[^ ^9^ ^]^ obtained from PlaqView^[^ ^10^ ^]^ (Figure S2a–e, Supporting Information).

### 2.2 DPP4 Expression on CD4^+^ T Cells is Upregulated in Patients with Atherosclerosis

**Figure 2:**
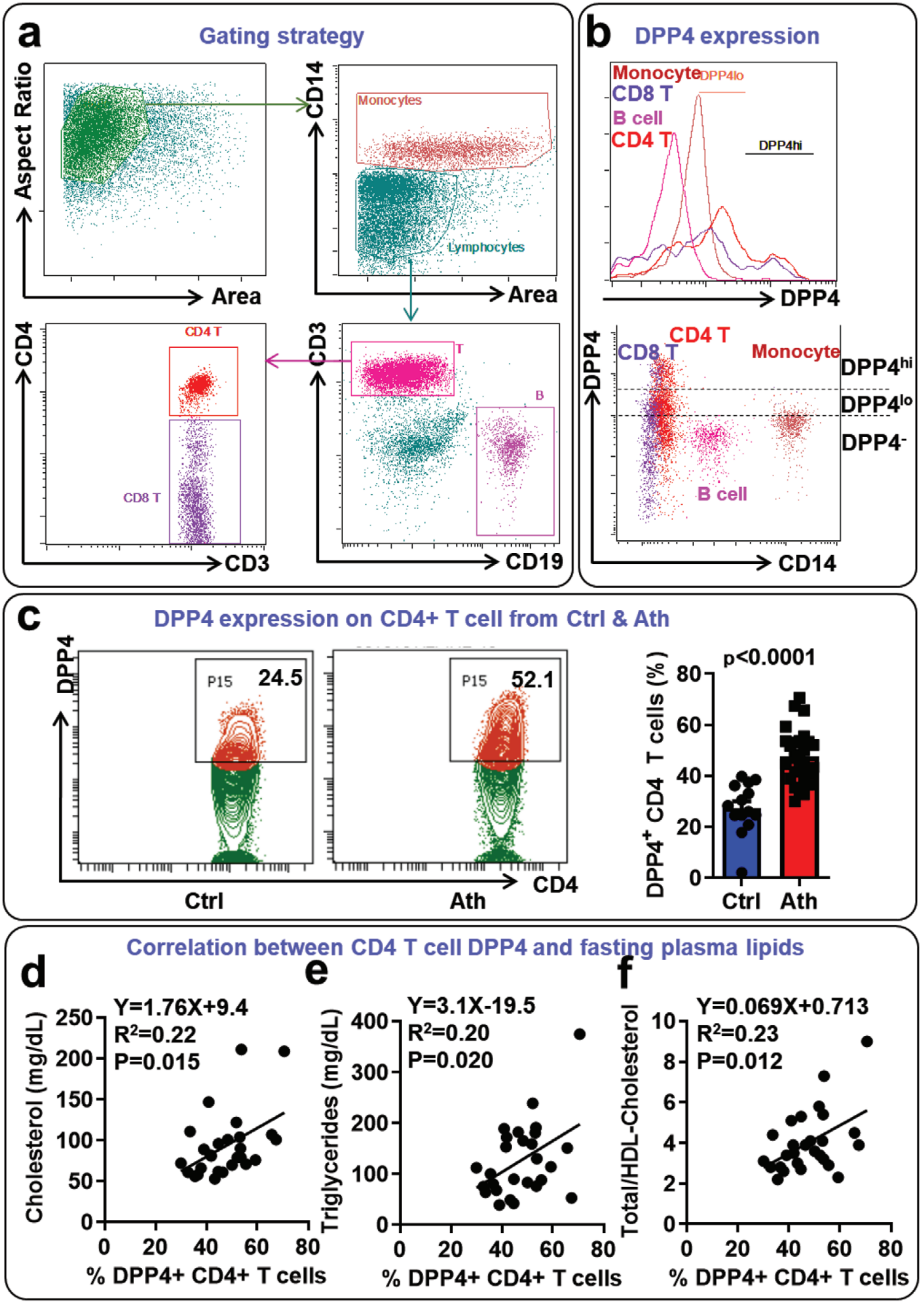
Dipeptidyl peptidase‐4 (DPP4) was increased in patients with atherosclerosis and was associated with plasma lipid levels: 27 patients with prior atherosclerotic disease and 14 healthy controls were recruited. a) Gating strategy of monocytes, B cells, CD4^+^ T cells, and CD8^+^ T cells. b) The expression of DPP4 on monocytes, B cells, CD4^+^ T cells, and CD8^+^ T cells in the blood of healthy controls. c) Flow cytometric detection of DPP4 expression on CD4^+^ T cells in patients with atherosclerosis (Ath) and controls (Ctrl). Representative contour plot (left panel) and statistical analysis (right panel) were shown. The means between the two groups was compared using unpaired Student's *t* test. d–f) Correlations between %DPP4^+^ cells in CD4^+^ T cells and levels of cholesterol (d), triglycerides (e), or ratio of total cholesterol/HDL‐cholesterol (f) were evaluated by linear regression. *N* = 27 for patients with atherosclerosis and *N* = 14 for healthy controls in figures a–c. *N* = 27 for figures d–f.

The single cell RNA sequencing data indicated that DPP4 was mainly expressed in cl.3 (CD4^+^ T cells) and was highly upregulated in CD4^+^ T cells in atherosclerosis. To confirm these findings at the protein level, we detected the expression of DPP4 on PBMCs from patients with atherosclerotic disease and control subjects by flow cytometry. The result shows that DPP4 was highly expressed on T cells, especially CD4^+^ T cells, which is consistent with the single cell RNA sequencing data. Monocytes expressed lower levels of DPP4, while B cells and granulocytes expressed minimal levels of DPP4 (**Figure** **2**a,b). Consistent with single cell RNA sequencing results, DPP4^+^ (including both DPP4^low^ and DPP4^high^) cells in CD4^+^ T‐cell population was significantly increased in patients with atherosclerosis (Figure 2c). In addition, the level of DPP4 expression on CD4^+^ T cells was positively correlated with plasma levels of cholesterol and triglycerides (Figure 2d–f), suggesting that the expression of DPP4 on T cells may be associated with dyslipidemia, which is a major risk factor for atherosclerosis.^[^ ^11^ ^]^ In contrast, the frequency of DPP4^+^ CD4^+^ T‐cell DPP4 was not associated with fasting blood glucose, fasting insulin, and statin therapy (Figure S3a–c, Supporting Information).

### 2.3 Contribution of Hematopoietic Cell Derived DPP4 to Systemic Glycemic Control

**Figure 3:**
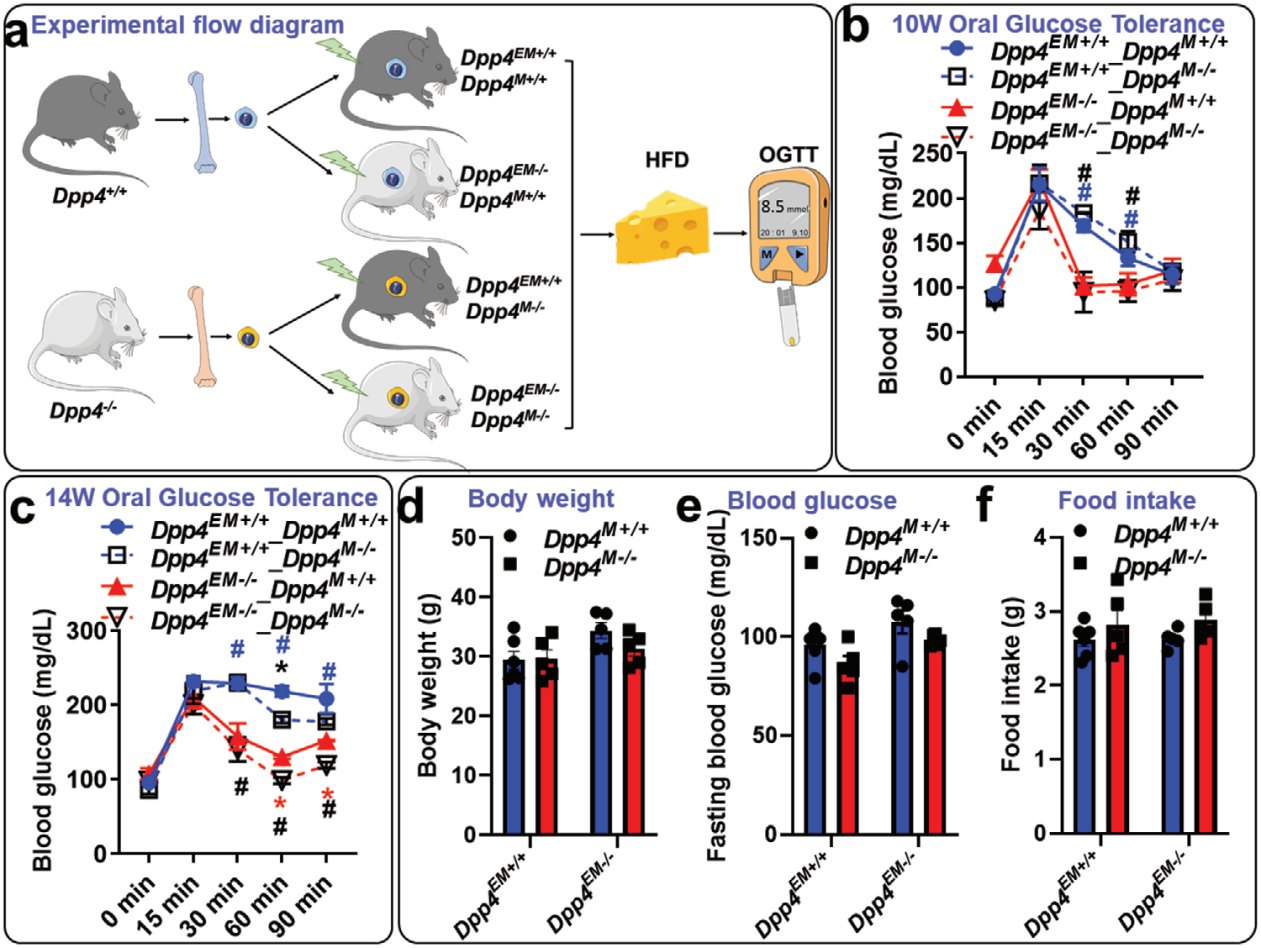
Contribution of bone marrow versus extramarrow tissue‐derived dipeptidyl peptidase‐4 (DPP4) to glucose intolerance: Irradiated *Dpp4^+/+^* (*Dpp4^EM+/+^*) or *Dpp4^−/−^* (*Dpp4^E−/−^*) mice were transplanted with bone marrow cells from *Dpp4^+/+^* (*Dpp4^M+/+^*) or *Dpp4^−/−^* (*Dpp4^M−/−^*) mice, followed by 14 weeks of high‐fat diet (HFD) (42% calories from fat) feeding. a) Diagram of reciprocal bone marrow transplantation was shown. b–f) Oral glucose tolerance tests at week 10 (b) or week 14 (c) after HFD feeding, body weight (d), fasting blood glucose (e), and food intake (f) were detected. *n* = 6 per group for *Dpp4^M+/+^* and 5 per group for *Dpp4^M−/−^*. Two‐way analysis of variance (ANOVA) analysis with Tukey post hoc test was used to compare the mean differences among the groups.^*^ *p* < 0.05 compared to *Dpp4^M+/+^*; ^#^ *p* < 0.05 compared to *Dpp4^EM+/+^*.

Since DPP4 can regulate the level of blood glucose through its enzymatic function and hyperglycemia is a risk factor for atherosclerotic disease, we assessed the contribution of hematopoietic and nonhematopoietic cell derived DPP4 to glucose metabolism. First, we examined the glucose regulation in the DPP4 whole‐body knockout and control mice. As with previously reported,^[^ ^12^ ^]^ the response to oral glucose tolerance test (OGTT) was improved in *Dpp4^−/−^* mice, when compared to *Dpp4^+/+^* controls (Figure S4a, Supporting Information). Improvement in insulin sensitivity in *Dpp4^−/−^* mice was confirmed by hyperinsulinemic‐euglycemic clamps, as indicated by a higher glucose infusion rate, insulin sensitivity index, and CLAMPed glucose disposal (Figure S4b–e, Supporting Information). Next, *Dpp4^+/+^* or *Dpp4^−/−^* bone marrow cells were transplanted into either *Dpp4^+/+^* or *Dpp4^−/−^* mice, followed by high‐fat diet (HFD) feeding for 14 weeks to test the role of hematopoietic cell versus extramarrow tissue derived DPP4 in blood glucose regulation. OGTT were performed at 10 weeks and 14 weeks of HFD. After 10 weeks of HFD, mice with a *Dpp4^−/−^* genotype of extramarrow tissues showed a significantly improved response to oral glucose challenge when compared to those with *Dpp4^+/+^* nonmyeloid tissues. In contrast, oral glucose responses were similar in mice with the same genotype in nonmarrow tissues, regardless of bone marrow genotype (**Figure** **3**a,b). Although mice with a *Dpp4^+/+^* bone marrow genotype had a slightly worse response to oral glucose challenge when compared to those with *Dpp4^−/−^* marrow genotype after 14 weeks of HFD, the glucose‐lowering effect of DPP4 deletion in nonmarrow tissue was more evident compared to that with bone marrow DPP4 deletion (Figure 3c). Both 10‐week and 14‐week glucose response data suggest that the majority of blood glucose lowering effect of DPP4 deletion originates from extramarrow tissues (Figure 3b,c). There were no significant differences in body weight, fasting blood glucose, food intake, and tissue weights between *Dpp4^+/+^* and *Dpp4^−/−^* chimeras (Figure 3d–f and Figure S5, Supporting Information).

### 2.4 Hematopoietic Cell‐Derived DPP4 is Involved in the Development of Diet‐Induced Atherosclerosis Progression

**Figure 4:**
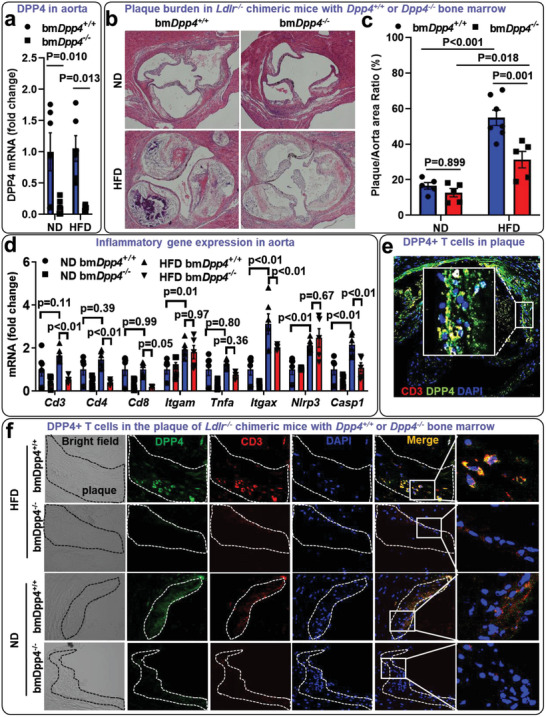
Hematopoietic deficiency of dipeptidyl peptidase‐4 (DPP4) reduced atherosclerosis and T‐cell mediated vascular inflammation in *Ldlr^−/−^* mice: *Ldlr^−/−^* mice transplanted with wild‐type (bm*Dpp4^+/+^*) or *Dpp4^−/−^* (bm*Dpp4^−/−^*) bone marrow were fed a high‐fat diet (HFD) or normal chow diet (ND) for 6 months. a) DPP4 expression in aortic tissue isolated from chimeric *Ldlr^−/−^* mice that were transplanted with *Dpp4^−/−^* bone marrow (bm*Dpp4^−/−^*) was compared with *Ldlr^−/−^* mice with *Dpp4^+/+^* bone marrow group (bm*Dpp4^+/+^*) by real‐time polymerase chain reaction (PCR) analysis. b,c) Aortic sinus plaque burden (b, representative images; c, statistical analysis) was detected by hematoxylin and eosin (H&E) staining. d) Real‐time PCR analysis of aortic tissues showed a reduction of T‐cell markers CD3, CD4, and CD8, but not macrophage (CD11b) and dendritic cell (CD11c) markers. e,f) Aortic sinus sections were stained with DAPI (blue), DPP4 (green), and CD3 (red), and imaged under a confocal microscope. Tiled image shows the infiltration of DPP4‐expressing T cells (yellow) in the plaque of HFD fed *Ldlr^−/−^* mice with *Dpp4^+/+^* bone marrow (e). Images showed a reduction of T‐cell infiltration in the plaque of *Ldlr^−/−^* mice with *Dpp4^−/−^* bone marrow (bm*Dpp4^−/−^*) when compared to *Ldlr^−/−^* mice with *Dpp4^+/+^* bone marrow (bm*Dpp4^+/+^*). f) Results were presented as mean ± SEM. Two‐way ANOVA analysis with Tukey post hoc test was used to compare the mean differences among the groups. *N* = 5–7 per group for figures a–f.

We next examined if hematopoietic cell derived DPP4 mediates atherosclerosis development. Irradiated atherosclerosis prone *Ldlr^−/−^* mice were transplanted with *Dpp4^+/+^* or *Dpp4^−/−^* bone marrow cells and fed a HFD or normal chow diet (ND) for 6 months. DPP4 expression in the aorta isolated from *Ldlr^−/−^* mice with *Dpp4^−/−^* bone marrow was markedly reduced when compared to the mice with *Dpp4^+/+^* bone marrow, suggesting that hematopoietic cells were the major source of DPP4 expression in the aorta of atherosclerotic mice (1 ± 0.43 versus 0.12 ± 0.05 for bm*Dpp4^+/+^* versus bm*Dpp4^−/−^*, *p* = 0.019; **Figure** **4**a). In consistency with reported studies,^[^ ^13^ ^]^ *Ldlr^−/−^* mice developed modest atherosclerosis lesions in the aortic root after 6 months ND feeding, while HFD‐fed *Ldlr^−/−^* mice with *Dpp4^+/+^* bone marrow showed extensive atherosclerotic lesions and higher levels of plasma cholesterol and triglycerides (Figure 4b,c, and Figure S6, Supporting Information). *Ldlr^−/−^* mice with *Dpp4^−/−^* bone marrow had smaller atherosclerotic plaques than those with *Dpp4^+/+^* bone marrow under HFD (50.69 ± 4.62% versus 30.12 ± 5.85% for HFD bm*Dpp4^+/+^* versus HFD bm*Dpp4^−/−^*, *p* < 0.001; Figure 4b,c), suggesting that hematopoietic deficiency of DPP4 reduced HFD‐induced atherosclerosis progression. T‐cell markers including *Cd3*, *Cd4*, and *Cd8* as well as the inflammatory gene *Casp1* were significantly decreased in the aorta isolated from HFD‐fed *Ldlr^−/−^* mice with *Dpp4^−/−^* bone marrow but not in ND‐fed mice. The expressions of macrophage maker *Itgam*, dendritic cell marker *Itgax*, and other inflammatory genes including *Tnfa* and *Nlrp3* were similar between mice with *Dpp4^+/+^* and *Dpp4^−/−^* bone marrow (Figure 4d). Confocal microscopy confirmed the presence of DPP4‐expressing T cells within the aortic plaque of both WT mouse (Figure 4e) and human (Figure S7, Supporting Information). Immunofluorescence staining showed a reduction of T‐cell infiltration in the atherosclerotic lesion of *Ldlr^−/−^* mice with *Dpp4^−/−^* bone marrow compared to that in mice with *Dpp4^+/+^* bone marrow under both ND and HFD (Figure 4f).

### 2.5 T‐Cell Specific Deficiency of DPP4 Reduces Atherosclerosis and T‐Cell Infiltration in the Aortic Tissue

**Figure 5:**
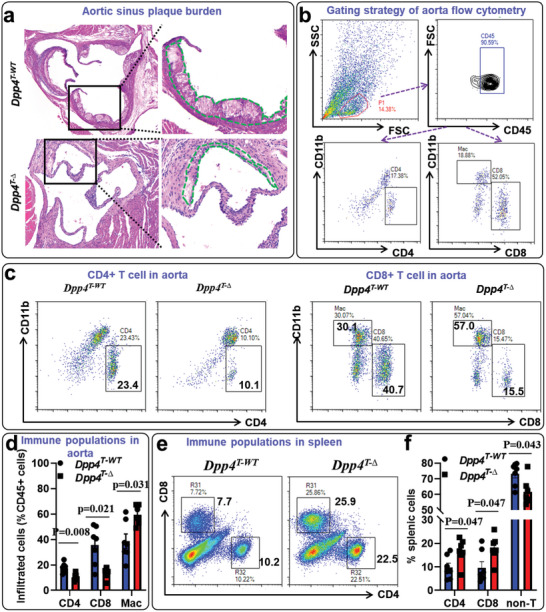
Deficiency of dipeptidyl peptidase‐4 (DPP4) on T‐cell reduced atherosclerotic lesion and T‐cell infiltration in aortic tissue: *Rag1^−/−^* mice lacking lymphocytes were adoptively transferred with T cells isolated from *Dpp4^−/−^* or *Dpp4^+/+^* mice to establish T‐cell specific DPP4 deficient mice (*Dpp4^T‐∆^*) and control mice (*Dpp4^T‐WT^*). The mice were then intravenously injected with 3 × 10^11^ vg AAV8 virus particles that overexpress proprotein convertase subtilisin/kexin type 9 (PCSK9), followed by 16 weeks of high‐fat diet (HFD) feeding to induce atherosclerosis. a, Section of aortic sinus were used for hematoxylin and eosin (H&E) staining to evaluate the plaque burden. b–f) Aorta and spleen tissues were harvested for the preparation of single cell suspension, followed by staining with CD45, CD11b, CD4, CD8, and DPP4. Stained cells were then analyzed on a flow cytometer to detect immune populations and DPP4 expression. CD4^+^ T cells, CD8^+^ T cells, and macrophages (CD11b^+^) were gated for further analyses (b). Representative dot plots (c) and statistical analysis (d) showed the frequencies of CD4^+^ T cells, CD8^+^ T cells, and macrophages in the aortic tissues of *Dpp4* ^T‐∆^ and *Dpp4* ^T‐WT^ mice. Representative dot plots (e) and statistical analysis (f) showed the frequencies of CD4^+^ T cells, CD8^+^ T cells, and non T cells (CD4^−^ CD8^−^) in the spleen of *Dpp4* ^T‐∆^ and *Dpp4* ^T‐WT^ mice. Two‐way ANOVA analysis with Tukey post hoc test was used to compare the mean differences among the groups. The data shown in this figure was representative of two independent experiments. *N* = 7 mice per group for figures a–f.

To test if T‐cell specific deficiency of DPP4 may reduce atherosclerosis, we adoptively transferred *Dpp4^+/+^* or *Dpp4^−/−^* pan T cells into lymphocyte deficient *Rag1^−/−^* mice in order to generate T‐cell specific DPP4 deficient chimeric mice. *Rag1^−/−^* mice with *Dpp4^+/+^* (*Dpp4^T‐WT^*) or *Dpp4^−/−^* T cells (*Dpp4^T‐∆^*) were then infected with adenoassociated viruses overexpressing proprotein convertase subtilisin/kexin type 9 (PCSK9) and fed a HFD to induce atherosclerosis.^[^ ^14^ ^]^ Flow cytometric detection of splenic cells confirmed that DPP4 was specifically deleted in CD4^+^ and CD8^+^ T cells, but not in CD3 cells (Figure S8a–e, Supporting Information). Atherosclerotic plaque burden in the aortic sinus was reduced in *Dpp4^T‐∆^* mice, when compared to that of *Dpp4^T‐WT^* mice (**Figure** **5**a). Aortic tissues were then digested to isolate aorta‐infiltrating immune cells. Flow cytometric analysis confirmed the deletion of DPP4 on aorta‐infiltrating T cells (Figure S9a–e, Supporting Information) and demonstrated a significant reduction of both CD4^+^ and CD8^+^ T cells in the aortas from *Dpp4^T‐∆^* mice (Figure 5b–d). In contrast, a larger amount of T cells were retained in the splenic reservoir in *Dpp4^T‐∆^* mice, compared to *Dpp4^T‐WT^* mice (Figure 5e,f). Quantitative real‐time polymerase chain reaction (PCR) analysis confirmed the reductions of T‐cell marker genes (*Cd3*, *Cd4*, and *Cd8*) in the aortas of *Dpp4^T‐∆^* mice (Figure S10a–c, Supporting Information), along with a decrease in marker genes of Th1 and Th17, but not Treg cells (Figure S10d–f, Supporting Information). Reduced Th1 and Th17 cytokines (IFN*γ* and IL‐17) in the atherosclerosis lesion were also observed in aortic sinus sections (Figures S11a,b, Supporting Information). No significant impact of DPP4 deficiency on T‐cell differentiation toward Th1 was observed (Figure S12a, Supporting Information), excluding differentiation defect as a cause for reduced Th1 in the atherosclerosis lesion of *Dpp4^T‐∆^* mice. Although the differentiations toward Th17 and Treg were not affected in *Dpp4^−/−^* T cells under unskewing (Th0) condition, DPP4 deficiency, but not pharmacological inhibition of DPP4 enzymatic activity, suppressed Th17 and Treg polarization under Th17 and Treg skewing conditions (Figure S12b–d, Supporting Information), suggesting a more complex mechanism regulating *Dpp4^−/−^* Th17 and Treg infiltration in plaque.

### 2.6 DPP4 Regulates T‐Cell Migration and Morphological Change

**Figure 6:**
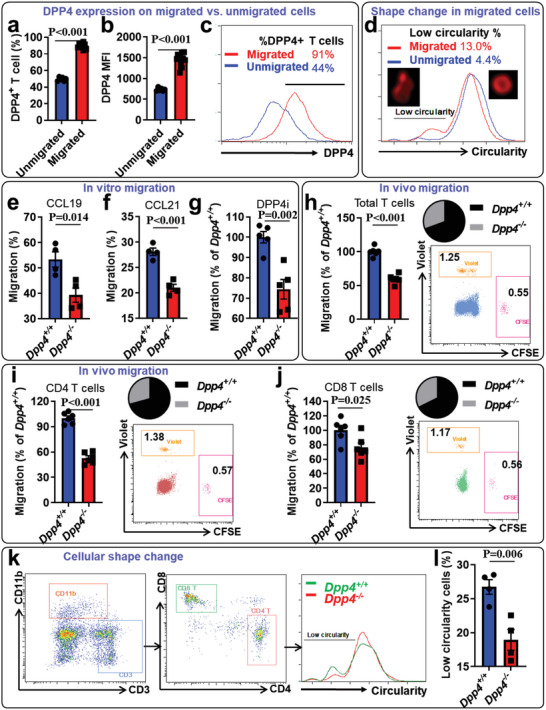
Loss of dipeptidyl peptidase‐4 (DPP4) reduced T‐cell migration and morphological changes: a–d) T cells isolated from wild‐type mice were used for a Transwell migration assay toward T‐cell chemokine CCL19. After 4‐h migration, T cells migrated into the bottom well (migrated) and those remained in the insert (unmigrated) were collected for imaging flow cytometric detection of DPP4 expression and morphology. a–c) Percentage of DPP4^+^ T cells (a), DPP4 mean fluorescence (MFI) (b), and representative histogram of DPP4 expression (c) were shown. Unpaired Student's *t* test was used to compare the mean differences between the groups. *N* = 6 for unmigrated group and 12 for migrated group. d) Representative histogram and images showing morphological changes in migrated and unmigrated cells. e,f) Migration of T cells isolated from *Dpp4^+/+^* or *Dpp4^−/−^* mice toward CCL19 (e) or CCL21 (f) was evaluated by Transwell migration assays. Unpaired Student's *t* test was used to compare the mean differences between the groups. *N* = 4 per group. g) Migration of *Dpp4^+/+^* or *Dpp4^−/−^* T cells toward CCL19 in the presence of DPP4 enzymatic inhibitor (DPP4i) alogliptin. Unpaired Student's *t* test was used to compare the mean differences between the groups. *N* = 5 per group. h–j) In vivo migration of *Dpp4^+/+^* and *Dpp4^−/−^* T cells. T cells isolated from *Dpp4^+/+^* or *Dpp4^−/−^* mice were labeled with CellTrace violet or CFSE, respectively and mixed at a ratio of 1:1. Mixed cells were then injected into the footpad of *Dpp4^+/+^* mice. After 12 h, popliteal lymph nodes were isolated and analyzed for T‐cell migration using flow cytometry. The migration of *Dpp4^+/+^* and *Dpp4^−/−^* total T cells (h), CD4^+^ T cells (i), and CD8^+^ T cells (j) into popliteal lymph nodes (left, statistical analysis of cell migration normalized by that in *Dpp4^+/+^* T cells; right, pie chart and representative dot plot showing the percentage of labeled *Dpp4^+/+^* or *Dpp4^−/−^* T cells in popliteal lymph nodes) were shown. *N* = 6 per group. k,l) *Dpp4^+/+^* and *Dpp4^−/−^* T cells were used for imaging flow cytometric detection of morphological change. Representative flow cytometric images (k) and statistical analysis of *Dpp4^+/+^* and *Dpp4^−/−^* T cells with low circularity (l) were shown. *N* = 4 per group. Results were presented as mean ± SEM. Unpaired Student's *t* test was used to compare the mean differences between the groups.

It has been well demonstrated that plaque‐infiltrating T cells originate from the recruitment of circulating T cells into the arterial walls.^[^ ^1^, ^15^ ^]^ To find out why T‐cell infiltration in the plaque was reduced in mice with *Dpp4^−/−^* T cells, we first performed in vitro migration assays and assessed the expression of DPP4 on migrated and unmigrated T cells. As a result, T cells with migratory activity displayed a higher expression of DPP4 as well as a higher level of morphological change, when compared to unmigrated T cells (**Figure** **6**a–d). Furthermore, *Dpp4^−/−^* T cells showed diminished migratory capacity in response to C‐C motif chemokine ligand 19 (CCL19) and C‐C motif chemokine ligand 21 (CCL21) gradients (Figure 6e,f). The expression levels of their receptor CCR7 were similar between *Dpp4^+/+^* and *Dpp4^−/−^* T cells (Figure S13, Supporting Information). In the presence of the DPP4 enzymatic inhibitor alogliptin, *Dpp4^−/−^* T cells still showed reduced levels of migration, further confirming the involvement of DPP4 nonenzymatic activity on T‐cell migration (Figure 6g). To confirm the in vivo role of DPP4 in the regulation of T‐cell migration, T cells isolated from *Dpp4^+/+^* and *Dpp4^−/−^* mice were labeled with CellTrace Violet and CFSE, respectively. The labeled cells were then mixed at a 1:1 ratio, followed by injection into the footpad of wild‐type mice. The migration of the labeled T cells to the popliteal lymph nodes was evaluated by imaging flow cytometry after 12 h. In line with the in vitro findings, fewer *Dpp4^−/−^* T cells migrated into lymph nodes (Figure 6h–j). Since changes in cell shape and cytoskeletal rearrangement are critical steps in cell adhesion and migration,^[^ ^16^ ^]^ we evaluated the shape of migrating and nonmigrating T cells. *Dpp4^−/−^* T cells had a lower prevalence of cells with a low circularity index and showed less chemokine‐driven F‐actin/*β*‐tubulin polarization compared to *Dpp4^+/+^* T cells (Figure 6k,l and Figure S14, Supporting Information). These results suggest that DPP4 may regulate both T‐cell migration and morphological changes. In addition to migration, local proliferation and apoptosis may also affect the number of T cells in local tissues. However, we did not observe significant differences in T‐cell proliferation (Figure S15, Supporting Information) and apoptosis (Figure S16, Supporting Information) between *Dpp4^+/+^* and *Dpp4^−/−^* mice.

### 2.7 DPP4 Deficiency Results in a Reduction in Mid1

**Figure 7:**
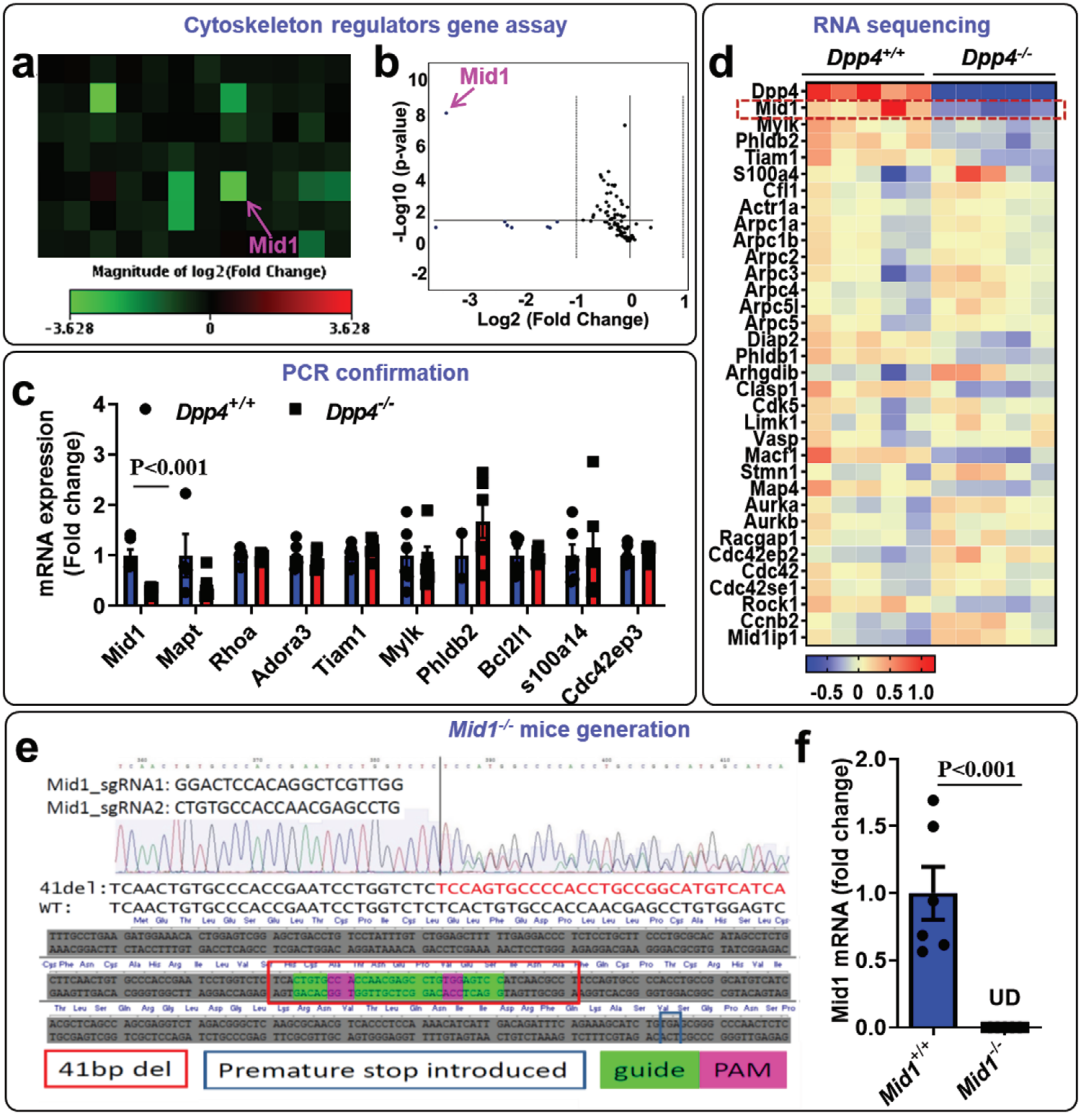
Potential involvement of Mid1 in dipeptidyl peptidase‐4 (DPP4) deficiency: a,b) Mouse cytoskeleton regulators gene array was performed using *Dpp4^+/+^* and *Dpp4^−/−^* T cells. Heat map (a) and volcano plot (b) were shown. The data was representative of two independent experiments. c) Real‐time polymerase chain reaction (PCR) confirmation of selected genes using *Dpp4^+/+^* and *Dpp4^−/−^* T cells. The downregulation of mid1 was confirmed in *Dpp4^−/−^* T cells. *N* = 6 per group. d) Heat map showing cytoskeleton regulatory gene expression using data collected from RNA sequencing of *Dpp4^+/+^* and *Dpp4^−/−^* mice. e) Sequence of guidance RNA for *Mid1^−/−^* generation and sequencing result for F1 *Mid1^+/−^* mice. f) Real‐time PCR results showing the absence of Mid1 mRNA in *Mid1^−/−^* mice. UD, undetectable. The difference between the two groups was compared using unpaired Student's *t* test. *N* = 6 per group.

Since cytoskeletal rearrangement is critical for cell migration,^[^ ^16^ ^]^ we performed a gene array to detect the expression of cytoskeleton regulators in *Dpp4^−/−^* T cells. Our results suggested that the expression of midline‐1 (Mid1) was significantly reduced in *Dpp4^−/−^* T cells (**Figure** **7**a,b). Real‐time PCR and RNA sequencing confirmed that *Mid1* expression was indeed reduced in *Dpp4^−/−^* mice (Figure 7c,d). To investigate the role of Mid1 in DPP4‐induced T‐cell migration, we generated a *Mid1^−/−^* mouse model using CRISPR‐cas9 technology, which resulted in a 41 base pair (bp) deletion in exon 2 of the *Mid1* gene resulting in a premature stop codon (Figure 7e). Real‐time PCR analysis confirmed the deficiency of Mid1 in *Mid1^−/−^* mice (Figure 7f).

**Figure 8:**
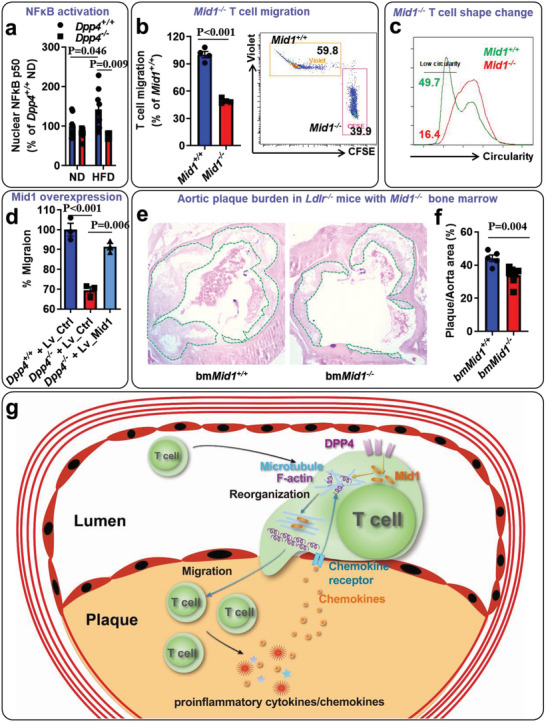
Deficiency of Mid1 reduced T‐cell motility and atherosclerosis: a) High‐fat diet (HFD) induced NF*κ*B activation in perivascular fat tissue was blunted in chimeric mice transplanted with *Dpp4^−/−^* bone marrow. Results were presented as mean ± SEM. Two‐way ANOVA analysis was used to compare the mean differences among the groups. *N* = 4–9 per group. b) Splenic T cells isolated from *Mid1^+/+^* or *Mid1^−/−^* mice were labeled with CellTrace Violet and CFSE, respectively. Cells were then mixed at 1:1 ratio and placed in the insert of a Transwell plate with CCL19 in the bottom well. Cells migrated to the bottom well and those remained in the insert were collected for flow cytometric detection. Migration was calculated based on the number of migrated and unmigrated cells (left panel). Representative dot plots show the percentage of *Mid1^+/+^* and *Mid1^−/−^* T cells in the bottom well of the Transwell plate (right panel). The difference between the two groups was compared using unpaired Student's *t* test. *N* = 4 per group. c) Circularity of *Mid1^+/+^* and *Mid1^−/−^* T cells in the bottom well (supplied with CCL19) of Transwell assay, as determined by imaging flow cytometry. d) *Dpp4^+/+^* T and *Dpp4^−/−^* T cells were infected with Mid1‐expressing or empty control lentivirus. Cells were then used for Transwell assay with CCL19 as the chemokine. Migrations normalized by *Dpp4^+/+^* T infected with control virus were calculated. Results were presented as mean ± SEM. Two‐way ANOVA analysis with Tukey post hoc test was used to compare the mean differences among the groups. e,f) *Ldlr^−/−^* mice transplanted with wild‐type (bm*Mid1^+/+^*) or *Mid1^−/−^* (bm*Mid1^−/−^*) bone marrow were fed a HFD for 16 weeks, aortic sinus plaque burden (e, representative images; f, statistical analysis) was reduced in mice reconstituted with *Mid1^−/−^* bone marrow. Plaque area was indicated by green dashed lines. Difference between the two groups was compared using unpaired Student's *t* test. *N* = 5 for bm*Mid1^+/+^* and 7 for bm*Mid1^−/−^* group. g) Schematic illustration of dipeptidyl peptidase‐4 (DPP4)/Mid1 axis in regulating T‐cell migration and atherosclerosis.

Mid1 has been reported to activate NF*κ*B in mouse bronchial epithelium by inhibiting protein phosphatase 2A (PP2A) activity.^[^ ^17^ ^]^ Using a TransAM NF*κ*B p50 assay kit (Active Motif), we found that NF*κ*B activity in the perivascular fat tissue was lower in *Ldlr^−/−^* mice reconstituted with *Dpp4^−/−^* bone marrow (**Figure** **8**a). We next investigated if *Mid1^−/−^* T cells show a similar migratory phenotype in Transwell migration assay. As depicted in Figure 8b and Figure S17 (Supporting Information), the migratory ability of *Mid1^−/−^* T cells was reduced to half of the *Mid1^+/+^* T cells. Similar to T cells deficient for DPP4, *Mid1^−/−^* T cells displayed fewer morphological changes and less chemokine‐driven F‐actin/*β*‐tubulin polarization (Figure 8c and Figure S14, Supporting Information), suggesting that T cells lacking DPP4 are analogous to deficiency in Mid1, both of which impair cytoskeletal rearrangement and T‐cell migration.^[^ ^18^ ^]^ Introducing exogenous Mid1 expression by a lentiviral vector largely restored migration in *Dpp4^−/−^* T cells (Figure 8d). In accordance with this, complement component 5*α*‐induced shape change was also markedly suppressed in macrophages deficient for Mid1 (Figure S18, Supporting Information). These results indicate that reduced expression of Mid1 is responsible for the low motility of *Dpp4^−/−^* T cells. In line with that, *Ldlr^−/−^* mice reconstituted with *Mid1^−/−^* bone marrow cells also showed a reduced atherosclerotic plaque burden when compared to *Ldlr^−/−^* mice with *Mid1^+/+^* bone marrow (Figure 8e,f), suggesting that Mid1 mediates the promotive effects of DPP4 on T‐cell migration and atherosclerosis (Figure 8g).

## 3 Discussion

DPP4 is well‐known for its glucose regulatory effects via enzymatic cleavage of incretin peptides such as GLP‐1, yet the role of DPP4 nonenzymatic activities in cardiometabolic disease has long been neglected. In this study, we reported a previously unrecognized role of DPP4 in atherosclerosis associated inflammation though regulation of T‐cell migration via Mid1, a microtubule‐associated protein. Defective DPP4/Mid1 signaling protected *Ldlr^−/−^* mice from HFD‐induced atherosclerosis. Collectively, we demonstrate that T‐cell derived DPP4 plays an important role in atherosclerosis by regulating Mid1‐mediated migration.

Chronic inflammation, involving both innate and adaptive immunity, has been demonstrated as an important pathogenic factor for the development of atherosclerosis.^[^ ^1^ ^]^ Both single cell RNA sequencing data and flow cytometric detection in our study showed a significant increase of DPP4 in circulating CD4^+^ T cells from patients with atherosclerosis. The expression level of DPP4 on CD4^+^ T cells was positively correlated with plasma levels of cholesterol and triglycerides, but not fasting blood glucose and insulin. This data indicates that DPP4 expressed on immune cells may be associated with dyslipidemia, an important risk of atherosclerosis. However, it should be noted that part of the patients were on lipid‐lowering therapy. We did not observe a significant difference in DPP4 expression between patients with and without statin therapy. It requires further investigation to examine whether lipid‐lowering drugs may affect DPP4 expression in T cells. Bone marrow transplantation on *Ldlr^−/−^* mice is widely used to study the role of blood‐borne immune cells in atherosclerosis.^[^ ^1b^ ^]^ In this study, transplantation of *Dpp4^−/−^* bone marrow into *Ldlr^−/−^* mice significantly suppressed HFD‐induced atherosclerosis progression, accompanied by a reduced vascular infiltration of T cells. In our study, no significant difference of *Cd3* and *Cd4* was observed in the aortas between ND‐ and HFD‐fed mice. This is probably because ND *Ldlr^−/−^* mice were 32 weeks old and had developed modest atherosclerosis lesion in the aorta root, a situation where the infiltration of the T cells in to the aortic wall had already occurred.^[^ ^1b^ ^]^ Unlike plaque‐infiltrating macrophages whose replenishment in the plaque predominantly depends on local proliferation,^[^ ^19^ ^]^ T cells within the plaque originate from the recruitment of circulating T cells into the arterial wall.^[^ ^1^, ^15^ ^]^ We also excluded the possibility of local proliferation and apoptosis by showing that DPP4 deficiency does not affect T‐cell proliferation and apoptosis. We then examined whether DPP4 is required for T‐cell migration. *Dpp4^−/−^* T cells displayed reduced migratory activity compared with *Dpp4^+/+^* T cells, even with the presence of DPP4 enzymatic inhibitor. These results suggest that DPP4 nonenzymatic activity regulates T‐cell migration. Adoptive transfer experiments in *Rag1^−/−^* mice using *Dpp4^+/+^* and *Dpp4^−/−^* T cells demonstrated reduced atherosclerosis and attenuated CD4^+^ and CD8^+^ T‐cell infiltration in mice with *Dpp4^−/−^* T cells.

DPP4 enzymatic activity has been shown to reduce both T‐cell and eosinophil migration to sites of tumor by inactivating the T‐cell chemokine CXCL10^[^ ^8b^ ^]^ and the eosinophil chemokine CCL11.^[^ ^8c^ ^]^ Here, we extended these findings by showing the nonenzymatic activity of DPP4 in cell migration. Therefore, DPP4 may regulate migration balance through both enzymatic and nonenzymatic forces. While enzymatic activity of DPP4 suppresses cell migration by degradation of chemokines, nonenzymatic activity promotes cell migration via upregulation of Mid1. Therefore, DPP4 appeared to play an important role in migratory capabilities of cell populations, particularly cells with high levels of DPP4 expression. Our results suggest an important dual role for DPP4 catalytic and noncatalytic functions in preserving migratory and homing function of T cells acting in concert with chemotactic signals.

Cytoskeletal rearrangement is a crucial step in the process of cell migration.^[^ ^16^, ^18^ ^]^ Actin filaments, microtubules, and intermediate filaments are the three main types of cytoskeletal polymers, which form an interconnected network, controlling the shape and mechanics of cell mobility through interaction with regulatory proteins. Here, we showed that DPP4 deficiency results in a reduction of Mid1, a key protein that binds to and regulates the function of microtubules.^[^ ^20^ ^]^ Mid1 interacts with the *α*4 regulatory subunit of the PP2A and mediates the ubiquitin‐dependent degradation of catalytic subunit PP2Ac.^[^ ^20^ ^]^ Additionally, Mid1 is involved in allergen‐induced asthma by inhibiting PP2A in bronchial epithelial cells.^[^ ^17^ ^]^ However, its role in vascular disease has yet to be elucidated. In this study, we found that Mid1 is responsible for DPP4‐mediated morphological change and migration in response to chemokine stimulation.

Bone marrow derived immune cells have been demonstrated to be an important source of systemic DPP4 activity from both the soluble form and the membrane‐bound form.^[^ ^21^ ^]^ To evaluate the extent to which bone marrow‐ and extramarrow tissue‐derived DPP4 contributes to glucose‐lowering effects, we generated bone marrow chimeric mice and detected responses to OGTT after 10 weeks or 14 weeks of HFD feeding. As demonstrated earlier by Wang et al., 10 weeks are sufficient to reconstitute systemic DPP4 enzymatic activity after bone marrow transplantation.^[^ ^21b^ ^]^ We observed minimal improvement in glucose tolerance in *Dpp4^−/−^* bone marrow‐transplanted animals after both 10 weeks and 14 weeks of diet feeding. In contrast, glycemic improvement was seen in mice with extramarrow DPP4 deficiency regardless of diet treatment duration. These results suggest that the incretin effect in *Dpp4^−/−^* mice was mainly attributable to extramarrow tissues and the improvement of atherosclerosis seen in *Ldlr^−/−^* mice with *Dpp4^−/−^* bone marrow may be unrelated to the glycemic effect of DPP4. This is consistent with previous studies reporting that many cell types within extramarrow tissues, such as hepatocytes and adipocytes, are major sources of circulating soluble DPP4.^[^ ^22^ ^]^ In addition, we also failed to find significant correlations between T‐cell DPP4 expression and levels of fasting blood glucose or insulin. This indicates that T‐cell DPP4 may have a minimal impact on the regulation of insulin and blood glucose levels. Interestingly, a slight glycemic improvement was observed after 14 weeks, but not 10 weeks of HFD feeding. Inflammation has been shown to induce insulin resistance with long‐term (e.g., 14 weeks), but not short‐term, HFD treatment.^[^ ^23^ ^]^ Therefore, the differing responses to glucose tolerance test at week 10 and 14 of HFD feeding are likely a result of DPP4 deficiency in bone marrow‐derived immune cells that alleviates obesity‐induced inflammation and insulin resistance at week 14. Considering the role of DPP4 in regulating T‐cell migration, it is reasonable to speculate that the improvement of OGTT at week 14 may be caused by the immunosuppressive role of DPP4 deficiency in inflammation. It has also been reported that circulating soluble DPP4 level is dissociated from the extent of metabolic inflammation,^[^ ^22^, ^24^ ^]^ which is consistent with our findings that bone marrow‐derived DPP4 promotes inflammation, while extramarrow DPP4 is responsible for incretin degradation.

In summary, this study identified a previously unrecognized role of DPP4 in regulating T‐cell migration and atherosclerosis. DPP4 controls T‐cell migration by regulating Mid1‐dependent cytoskeletal rearrangement, and thus promotes vascular inflammation in atherosclerosis. We acknowledge several limitations in this study. First, the precise mechanisms by which DPP4 regulates Mid1 expression were not fully investigated. We have obtained preliminary evidence showing epigenetic modification is involved in this process (data not shown), which will be further addressed in subsequent studies. Another limitation is that we cannot definitely exclude the involvement of other DPP4‐expressing immune cells. Although T cells are the predominant population that expresses DPP4 among immune cells and we confirmed the involvement of T‐cell derived DPP4 in atherosclerosis by using T‐cell adoptive transfer experiment, the involvement of other DPP4‐expressing cells such as macrophages and dendritic cells could not be excluded. Future studies using cell‐specific DPP4 knockout models will be necessary to examine the role of DPP4 in other types of cells and atherosclerosis.

## 4 Experimental Section

### Study Participants and Ethical Approval

All procedures related to human participants in this study were approved by the Institutional Review Board (IRB) at The Ohio State University and Case Western Reserve University. All participants have provided a written informed consent before participating in this study. Twenty‐seven patients with previously documented atherosclerotic cardiovascular disease enrolled in the ALPINE trial, a phase 4 clinical trial (ClinicalTrials.gov Identifier: NCT01417104),^[^ ^25^ ^]^ were included in this study. A total of 14 age‐ and sex‐matched individuals without documented cardiovascular or metabolic diseases were used as healthy controls. Atherosclerotic cardiovascular disease was defined as one or more of the following conditions: previous coronary artery bypass graft surgery, cerebrovascular accident, myocardial infarction, peripheral arterial disease (ankle‐brachial index < 0.9 and/or prior peripheral intervention or surgery), and/or percutaneous coronary intervention (Table S1, Supporting Information). Exclusion criteria included: history of malignancy, diagnosis of type 1 diabetes or use of hypoglycemic drugs, uncontrolled hypertension (>145/90 mmHg); renal insufficiency defined as glomerular filtration rate < 40 mL min^−1^ (derived with the Modified Diet in Renal Disease equation). The presence of thoracic aortic atherosclerosis in patients with atherosclerotic cardiovascular disease was confirmed by a high‐resolution 3D MRI scanning with fat suppressed, dark blood, turbo spin echo sequence with variable flip angles as described previously.^[^ ^25^ ^]^ The human aorta was obtained from a patient who had an abdominal aortic aneurysm requiring vascular replacement. The tissues were immediately placed in cold 4% paraformaldehyde buffer after removal from the patients. A consent was obtained before the surgery.

### Animals and Reagents

*Ldlr^−/−^* and wild‐type C57BL/6 mice were purchased from Jackson Laboratory. *Rag1^−/−^* mice were purchased from GemPharmatech Co., Ltd (Jiangshu, China). DPP4 knockout (*Dpp4^−/−^*) mice on C57BL/6 background^[^ ^26^ ^]^ were a generous gift from Dr. Didier Marguet at the French National Institute for Health and Medical Research and were bred in SPF animal facilities using heterozygous x heterozygous breeding pairs. Resulting homozygous *Dpp4^−/−^* and wild‐type (*Dpp4^+/+^*) littermates were used for experiments. *Foxp3‐GFP* mice on C57BL/6 background (Strain #: 0 06772) that coexpress EGFP and Foxp3 under the control of the endogenous promoter were purchased from the Jackson Laboratory. *Dpp4^−/−^* mice were cross with *Foxp3‐GFP* mice to generate *Foxp3‐GFP Dpp4^−/−^* mice. All procedures in the animal studies were reviewed and approved by the IACUC committee at The Ohio State University, Case Western Reserve University, and Tongji Hospital affiliated to Tongji Medical College of Huazhong University of Science and Technology.

*Mid1^−/−^* mice were generated using CRISPR‐Cas9 technology by contracting with The Jackson Laboratory. In brief, two single guide RNAs (sgRNA) targeting exon 2 of the Mid1 coding sequence and wild‐type Cas9 were injected into C57BL/6J zygotes to introduce frameshift mutations, followed by embryo transfer into pseudopregnant female mice. The sgRNA sequences are as follows: Mid1_sgRNA1: GGACTCCACAGGCTCGTTGG; Mid1_sgRNA2: CTGTGCCACCAACGAGCCTG. The successful generation of frameshift mutations was determined by gene sequencing and genotyping. A total of 5 founders with frameshift mutations were generated (Table S2, Supporting Information).

All antibodies used for flow cytometry were purchased from eBioscience (San Diego, CA), BD Biosciences (Franklin Lakes, NJ), R&D systems (Minneapolis, MN), or Biolegend (San Diego, CA). CCL19 and CCL21 used in the migration assays were purchased from R&D systems (Minneapolis, MN). The cytoskeleton regulators RT^2^ Profiler PCR Array was from QIAGEN (Germantown, MD) and mRNA sequencing was performed by Novogene (Sacramento, CA).

To induce atherosclerosis in *Ldlr^−/−^* mice, animals were fed an atherogenic HFD (42% calories from fat, Harlan TD.88137) or normal chow diet (ND) for 4–6 months. To induce atherosclerosis in *Rag1^−/−^* mice adoptively transferred with WT or *Dpp4^−/−^* T cells, mice were infected with adeno‐associated virus serotype 8 (AAV8) overexpressing proprotein convertase subtilisin/kexin type 9 (PCSK9) and fed a HFD for 16 weeks.

### Single Cell RNA Sequencing

Anticoagulated peripheral blood collected from patients with atherosclerotic disease or matched healthy controls were used for the isolation of PBMCs by density gradient centrifugation using Ficoll as previously described.^[^ ^21c^ ^]^ Isolated PBMCs were loaded into single‐cell gel beads and barcoded with unique molecular identifier using the single cell 3’ reagent kit (10× Genomics) as instructed by the manufacturer's protocol. The complementary DNA (cDNA) was then generated, libraries were constructed, and sequenced on a NovaSeq platform (Illumina). The data was analyzed by Loupe Browser (10× Genomics) and SeqGeq Software (FlowJo). For the validation, single cell RNA sequencing datasets from PlaqView^[^ ^10^ ^]^ were reanalyzed to detect the expression of *Dpp4* in human aorta/plaque‐infiltrating cells from patients with atherosclerosis,^[^ ^9a^ ^]^ aortic aneurysm, and control subjects.^[^ ^9b^ ^]^

### Gene Array and RNA Sequencing

Total RNA was isolated using an RNeasy Mini Kit (QIAGEN). DNA was quantified using NanoDrop (Thermo Scientific NanoDrop 2000) and the integrity of RNA was checked by agarose gel electrophoresis. The expression of cytoskeleton regulator genes was determined using a Mouse Cytoskeleton Regulators RT^2^ Profiler PCR Array (QIAGEN) according to the manufacturer's instruction. For RNA sequencing, total RNA was sent to Novogene for sequencing on an Illumina NovaSeq platform and analyzed as described earlier.^[^ ^27^ ^]^

### Hyperinsulinemic‐Euglycemic Clamp

The hyperinsulinemic‐euglycemic clamp experiment was performed as previously described.^[^ ^28^ ^]^ Briefly, jugular vein‐cannulated mice were acclimated to restrainers (3 h per day for 3 days) before the clamp experiment. After fasting for 5 h, [3‐^3^H]‐glucose was infused at 0.05 µCi min^−1^ for 120 min and 20 µL blood samples were collected at 90, 100, and 110 min after the initiation of infusion to access basal hepatic glucose production. The animals were then primed with 60 mU kg^−1^ regular human insulin (Novo Nordisk), followed by a constant infusion at 2.5 mU kg^−1^ min^−1^. During insulin infusion, blood glucose levels were measured every 10 min and a variable infusion of 25% dextrose was adjusted to maintain the blood glucose at 120–130 mg dL^−1^. Blood samples were collected at 100, 110, and 120 min during insulin infusion. Insulin sensitivity index, glucose infusion rate, and basal/clamped glucose disposal were calculated as described previously.^[^ ^28^ ^]^

### Oral Glucose Tolerance Test

For oral glucose tolerance test, baseline blood glucose, and body weights of animals were measured after overnight fasting with free access to drinking water. Mice were administered with 2.0 g kg^−1^ body weight D‐glucose by oral gavage and blood glucose levels were measured at 30, 60, 90, and 120 min after gavage using a Bayer Contour glucometer.

### Lentiviral Transduction of T Cells

For the transduction of T cells with Mid1‐overexpressing lentiviral particles (GeneCopoeia), 5 × 10^5^ primary T cells isolated from the spleen were resuspended in 100 µL concentrated viral supernatant at a multiplicity of infection (MOI) of 10, in the presence of 8 ng mL^−1^ polybrene, followed by a spinoculation at 1200 × *g* for 2 h at room temperature. After spinoculation, cells were washed with PBS and cultured at a concentration of 1 × 10^6^ cells mL^−1^ in RPMI 1640 containing 10% FBS for 72 h. Cells were then used for Transwell migration assay.

### Bone Marrow Transplantation

For bone marrow transplantation, 8‐week old male *Ldlr^−/−^* mice were irradiated at a dose of 950 Rad. The mice were then reconstituted with 15 × 10^6^ bone marrow cells isolated from WT, *Dpp4^−/−^*, or *Mid1^−/−^* mice 24 h after irradiation, as described previously.^[^ ^29^ ^]^ Two weeks after bone marrow reconstruction, the chimeric mice were fed HFD or ND for a duration of 16–24 weeks as indicated.

### Quantification of Atherosclerosis Lesions

The heart and arteries were embedded in paraffin and 10 µm cross‐sections with three aortic valves present were collected. Hematoxylin and eosin (H&E) staining of the aortic sinus section was used to quantify atherosclerotic plaque size. The lesion area and aortic lumen area in the H&E sections was measured using ImageJ software. The ratio of lesion area/lumen area of each animal was determined by calculating the mean lesion/lumen ratio of three sinus sections at 60 µm intervals.

### Confocal Microscopy

For the detection of DPP4 and T‐cell marker CD3 in aortic plaque, paraffin‐embedded sections of aortic sinus were used for immunofluorescence staining after antigen retrieval with 1× antigen retriever buffer (Sigma–Aldrich). In brief, 10‐µm‐thick tissue sections were incubated with primary antibodies (anti‐mouse CD3 and anti‐mouse DPP4) at 4 °C overnight. After washes and incubating with corresponding secondary antibodies, sections were mounted in Fluormount G (Thermo Fisher). Images were captured on a Zeiss LSM 510 Confocal Laser Scanning Microscope (Carl Zeiss) and analyzed using LSM Image Browser (Carl Zeiss).

### In Vitro and In Vivo Migration Assays

In vitro cell migration was evaluated by an in vitro Transwell assay using Corning Transwell 24‐well plates (6.5 mm diameter, 5 × 10^−6^ m pore) as described previously.^[^ ^30^ ^]^ Briefly, 5 × 10^5^ fluorescently labeled or unlabeled splenocytes in 100 µL cell culture medium (RPMI‐1640 containing 10% fetal bovine serum) were placed in the insert (upper chamber) of a Transwell plate, with the lower chamber filled with 600 µL cell culture medium containing indicated chemokines (0.5 µg mL^−1^ CCL19 or 1 µg mL^−1^ CCL21). After a 5‐h incubation in a 37 °C CO_2_ incubator, cells from both the upper and the lower chambers were collected for cell counting and flow cytometric analysis after staining with cell population markers (such as anti‐mouse CD3e, CD4, and CD8).

For in vivo migration assay, 1 × 10^6^ CellTrace CFSE‐labeled *Dpp4^−/−^* T cells and CellTrace violet‐labeled *Dpp4^+/+^* T cells were mixed at 1:1 ratio in 20 µL 1× PBS. Cells were then injected into the foot pad of a C57BL/6 mouse. Popliteal lymph nodes were isolated for the preparation of single cell suspension 12 h after injection. Isolated cells were then counted and stained with anti‐mouse CD3e, CD4, and CD8, followed by flow cytometric analysis.

### Flow Cytometry and Immunofluorescence Microscopy

Imaging flow cytometric detection was performed on an Amnis Flowsight (Millipore Sigma, Seattle, WA). T‐cell morphology was measured by imaging flow cytometry and immunofluorescence microscopy. For imaging flow cytometric detection of morphology, circularity, defined as average cellular radius divided by the variance in the radii, was used to quantify the cellular morphology as described.^[^ ^31^ ^]^ For immunofluorescence microscopic detection of morphology and polarization, wild‐type, *Dpp4^−/−^*, and *Mid1^−/−^* T cells were seeded on 2 µg mL^−1^ mouse ICAM‐1 coated slides and treated with 0.5 µg mL^−1^ CCL19 or PBS for 10 min. After fixation and permeabilization, cells were stained with iFluor 555 Phalloidin (for the detection of F‐actin) and rabbit anti‐mouse *β*‐tubulin, followed incubation with Alexa Fluor 488 donkey anti‐rabbit second antibody). Images were captured on a fluorescence microscope (MshOt, Guangzhou, China) and analyzed using ImageJ software (NIH, Bethesda, MD, USA). Conventional flow cytometry was performed on a LSRII cytometer (BD Biosciences, San Jose, CA). All flow cytometry antibodies were purchased from BioLegend (San Diego, CA), BD, Invitrogen (Waltham, MA), or R&D Systems (Minneapolis, MN). Cells were stained following standard procedure. For the evaluation of cell morphology, flow data collected from Amnis Flowsight were analyzed by IDEAS 6.2 software as instructed by the manufacturer.

### Real‐Time PCR

Total RNA was extracted from cells or tissue samples using Trizol Reagent (Life Technologies, Grand Island, NY) according to the manufacturer's instruction. High Capacity cDNA Reverse Transcriptase Kit (Life Technologies) was used to synthesize cDNA from RNA. The quantitative real‐time PCR amplification of target genes was performed on a LightCycler 480 real‐time PCR System (Roche Applied Science, Indianapolis, IN) using a LightCycler 480 SYBR Green I Master kit (Roche Applied Science). mRNA expression was normalized to *β*‐actin and ΔΔ*C*_t_ method was used to determine the expression level.

### Statistical Analysis

All data are presented as mean ± standard error of the mean (SE). Graphpad Prism (version 8.0) was used for statistical analysis and graphing. Student's *t* test was used to detect the differences between two groups, such as DPP4 positive CD4 T‐cell frequencies between patients with atherosclerosis and healthy controls, DPP4 expression levels between migrated and unmigrated cells, migration between *Dpp4^−/−^* and *Dpp4^+/+^* cells or between *Mid1^−/−^* and *Mid1^+/+^* cells, and plaque area between *Ldlr^−/−^* mice with *Mid1^−/−^* and *Mid1^+/+^* bone marrow. The differences of OGTT response among the groups were tested by two‐way analysis of variance (ANOVA) with Tukey post hoc test. One‐way ANOVA with Tukey post hoc test was used to determine the differences between the means of three or more independent groups. Linear regression was used to evaluate the associations between DPP4 expression and plasma triglyceride, cholesterol, fasting blood glucose, or fasting insulin levels. A *p* value of <0.05 was considered statistically significant.

## Conflict of Interest

The authors declare no conflict of interest.

## Author Contributions

X.R., Y.W., Q.G., J.C., and J.Z. researched data. G.M. was responsible for human MRI assessment of plaque. G.Z. was responsible for the human aortic tissue collection from the patients. X.J.S and M.J.Q. performed the CLAMP studies. X.R. and J.Z. wrote the manuscript. M.R., M.B.F., Z.B., Z.L., M.J.Q., S.R., and J.Z. reviewed and edited the manuscript. M.B.F., X.J.S., M.J.Q., L.D., and S.I.T. contributed to discussion of experimental design.

## Supporting information

Supporting Information

## Data Availability

The data that support the findings of this study are available on request from the corresponding author. The data are not publicly available due to privacy or ethical restrictions.
