# Supplementary material for: Dipeptidyl Peptidase 4/Midline‐1 Axis Promotes T Lymphocyte Motility in Atherosclerosis
Source: Adv Sci (Weinh). 2023 Jan 22;10(9):2204194. doi: 10.1002/advs.202204194 (PMC10037965; doi:10.1002/advs.202204194)
Supplement: Supplementary file 1 — Supporting Information [file ADVS-10-2204194-s001.pdf]

ADVANCED SCIENCE
Open Access

Supporting Information

for *Adv. Sci.*, DOI 10.1002/advs.202204194

Dipeptidyl Peptidase 4/Midline-1 Axis Promotes T Lymphocyte Motility in Atherosclerosis

*Xiaoquan Rao, Michael Razavi, Georgeta Mihai, Yingying Wei, Zachary Braunstein, Matthew B. Frieman, Xiao Jian Sun, Quan Gong, Jun Chen, Gang Zhao, Zheng Liu, Michael J. Quon, Lingli Dong, Sanjay Rajagopalan and Jixin Zhong**

Supplementary Tables

Table S1 Baseline characteristics of patients

Characteristic	Total (N=27)
Age - years: Mean (SD)	60.9 (8.3)
Race	
White: No. (%)	22 (81.5)
Black: No. (%)	5 (18.5)
Medical history of CVD	
History of Myocardial Infarction: No. (%)	11 (40.7)
Coronary stent implantation: No. (%)	14 (51.9)
Coronary artery bypass grafting: No. (%)	7 (25.9)
Stroke: No. (%)	2 (7.4)
Peripheral arterial disease: No. (%)	8 (29.6)
Medication	
ACE/ARB: No. (%)	15 (55.6)
Statin: No. (%)	17 (63.0)
Aspirin: No. (%)	19 (70.4)
Beta Blockers: No. (%)	19 (70.4)
CCB: No. (%)	2 (7.4)

Table S2 Mid1 mutations generated by Crispr-Cas9 technology

#	Gender	Date of Birth	Result
7824	Male	8/2/2016	4bp deletion
7825	Male	8/2/2016	17bp deletion
7831	Male	8/2/2016	41bp deletion
7832	Male	8/2/2016	1bp insert
7839	Male	8/2/2016	5bp deletion

Supplementary Figures and Figure Legends

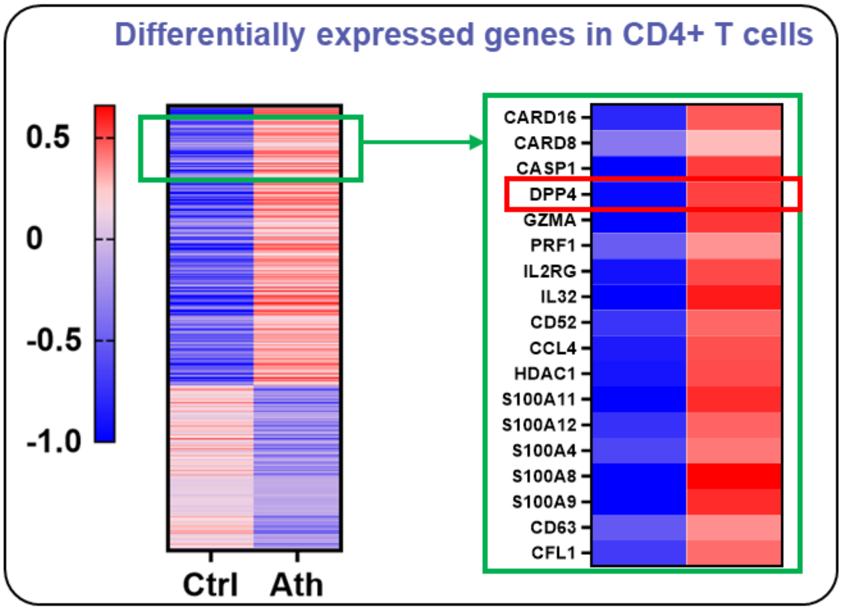

Fig. S1 Differentially expressed genes in CD4$^+$ lymphocytes between atherosclerosis and healthy control: Heatmap shows the differentially expressed genes in CD4$^+$ lymphocytes from controls and patients with atherosclerosis.

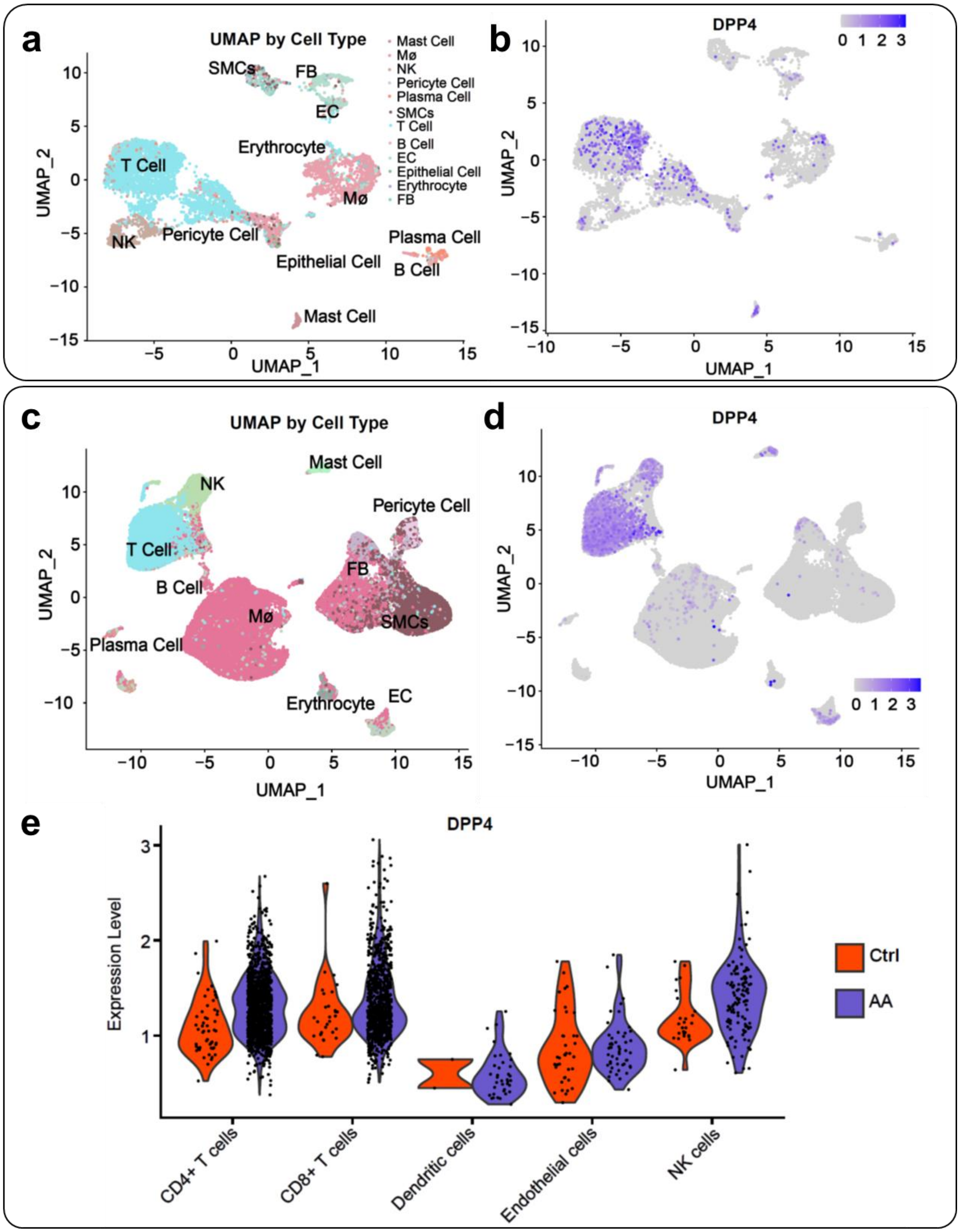

Fig. S2 Single cell RNA sequencing analyses of DPP4 expression from public datasets. a & **b**, UMAP plots showing clusters by cell type (**a**) and DPP4 expression (**b**) using single cell RNA sequencing dataset of human atherosclerotic plaques shared by Slenders et al.. **c** - **e**, UMAP plots showing clusters by cell type (**c**) and DPP4 expression (**d**) and violin plot showing the DPP4 expression in different cell subsets from patients

with aortic aneurysm or control subjects (**e**) using single cell RNA sequencing dataset of human aortic tissues shared by Li et al..

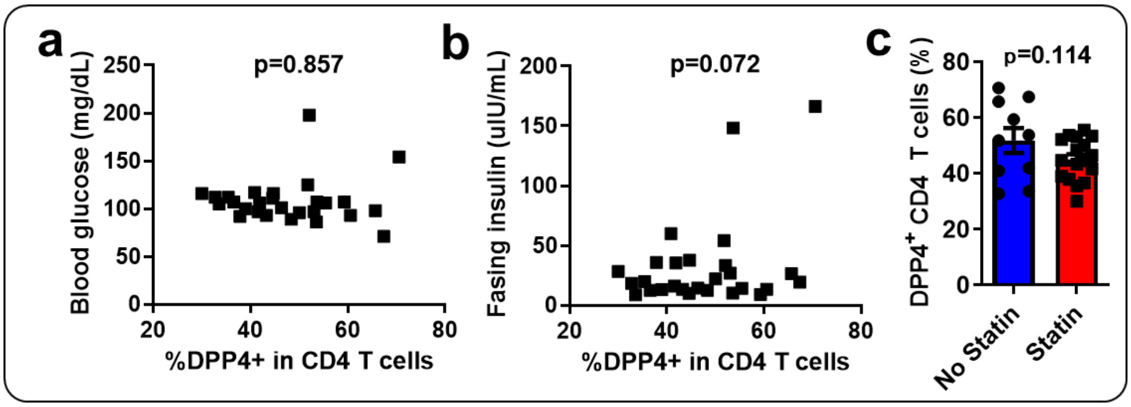

Fig. S3 DPP4 expression on CD4^{+} T cells does not correlate with fasting blood glucose and insulin levels: a, The scatter plot of CD4^{+}DPP4^{+} T cell percentage and fasting blood glucose level. **b**, The scatter plot of CD4^{+}DPP4^{+} T cell percentage and fasting plasma insulin level. **c**, DPP4 expression on CD4+ T cells from patients with or without statin therapy.

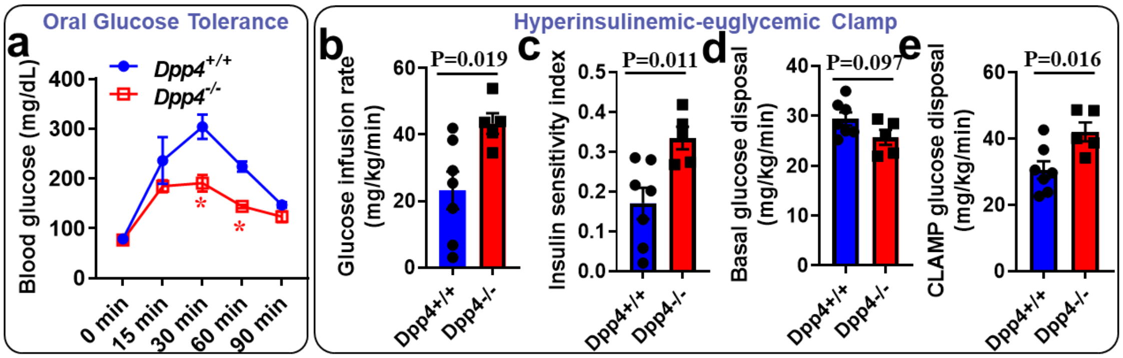

Fig. S4 Improved glucose intolerance in DPP4 knockout mice: a, Glucose tolerance test using oral glucose gavage (2g D-glucose/kg body weight). **b-e,** *Dpp4$^{+/+}$* and *Dpp4$^{-/-}$* mice were subjected to hyperinsulinemic euglycemic clamp measurement. Glucose infusion rate (**b**), insulin sensitivity index (**c**), basal glucose disposal (**d**), and CLAMP glucose disposal (**e**) rates were shown. n = 4-7 mice/group.

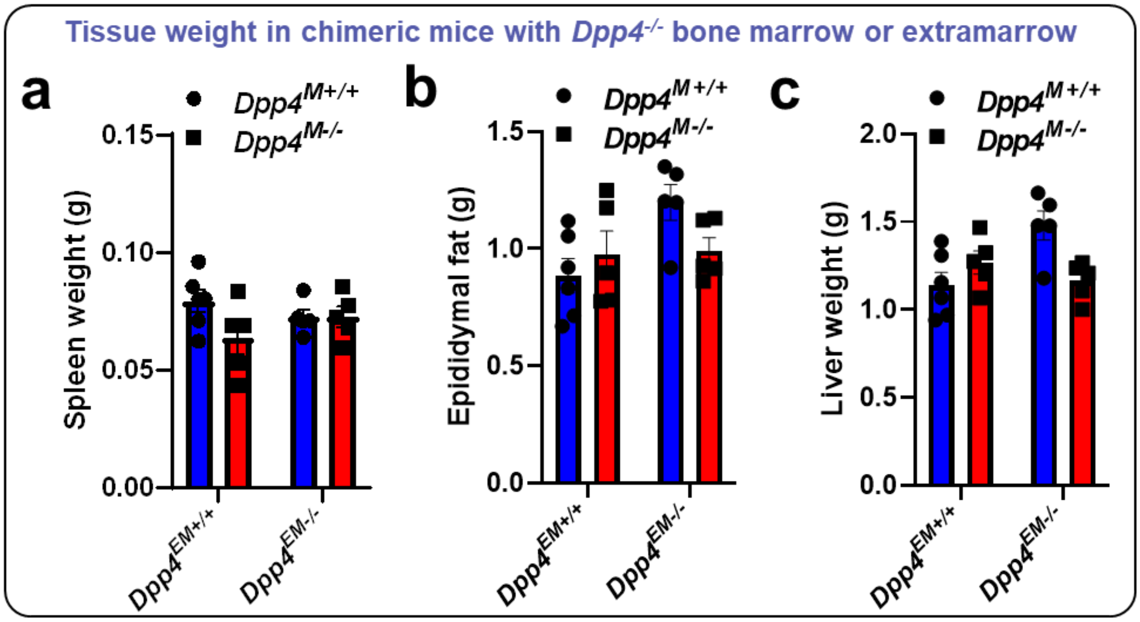

Fig. S5 Effects of bone marrow- or extramarrow-specific DPP4 deficiency on tissue weight: Irradiated *Dpp4*$^{+/+}$ (*Dpp4*$^{EM+/+}$) or *Dpp4*$^{-/-}$ (*Dpp4*$^{EM\,-/-}$) mice were transplanted with bone marrow cells from *Dpp4*$^{+/+}$ (*Dpp4*$^{M+/+}$) or *Dpp4*$^{-/-}$ (*Dpp4*$^{M\,-/-}$) mice, followed by 14 weeks of high fat diet (42% calories from fat) feeding. The weights of spleen (**a**), epididymal fat (**b**), and liver (**c**) were detected. n = 4-7 mice/group.

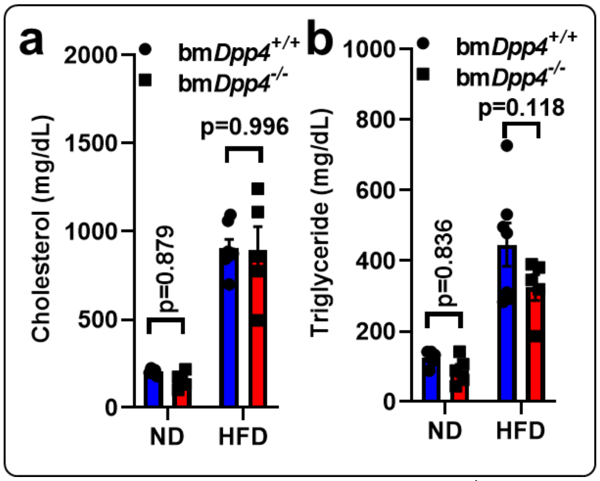

Fig. S6 Plasma cholesterol and triglycerides in *Ldlr*$^{-/-}$ mice with or without DPP4 deficiency in bone marrow-derived cells: *Ldlr*$^{-/-}$ mice transplanted with wild-type (bm*Dpp4*$^{+/+}$) or *Dpp4*$^{-/-}$ (bm*Dpp4*$^{-/-}$) bone marrow were fed a high fat diet (HFD) or normal chow diet (ND) for 6 months. Plasma was then collected for the detection of cholesterol (**a**) and triglycerides (**b**). N=5-7 mice/group.

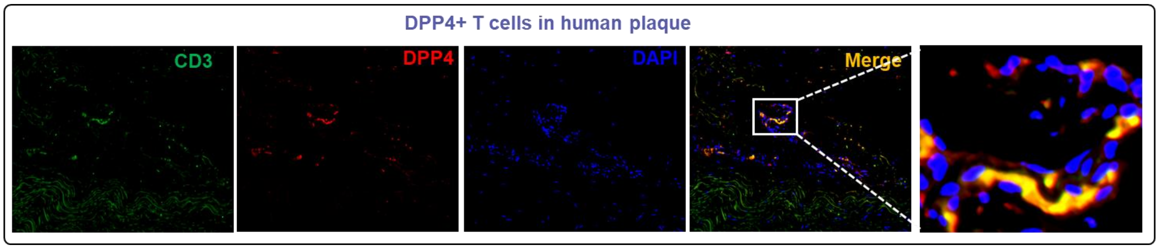

Fig. S7 DPP4 expressing T cells in human atherosclerosis plaque: Human plaque section was stained with anti-human CD3 and anti-human DPP4 antibodies and their corresponding fluorescently labeled secondary antibodies, followed by DAPI staining. Images were captured under a fluorescence microscope.

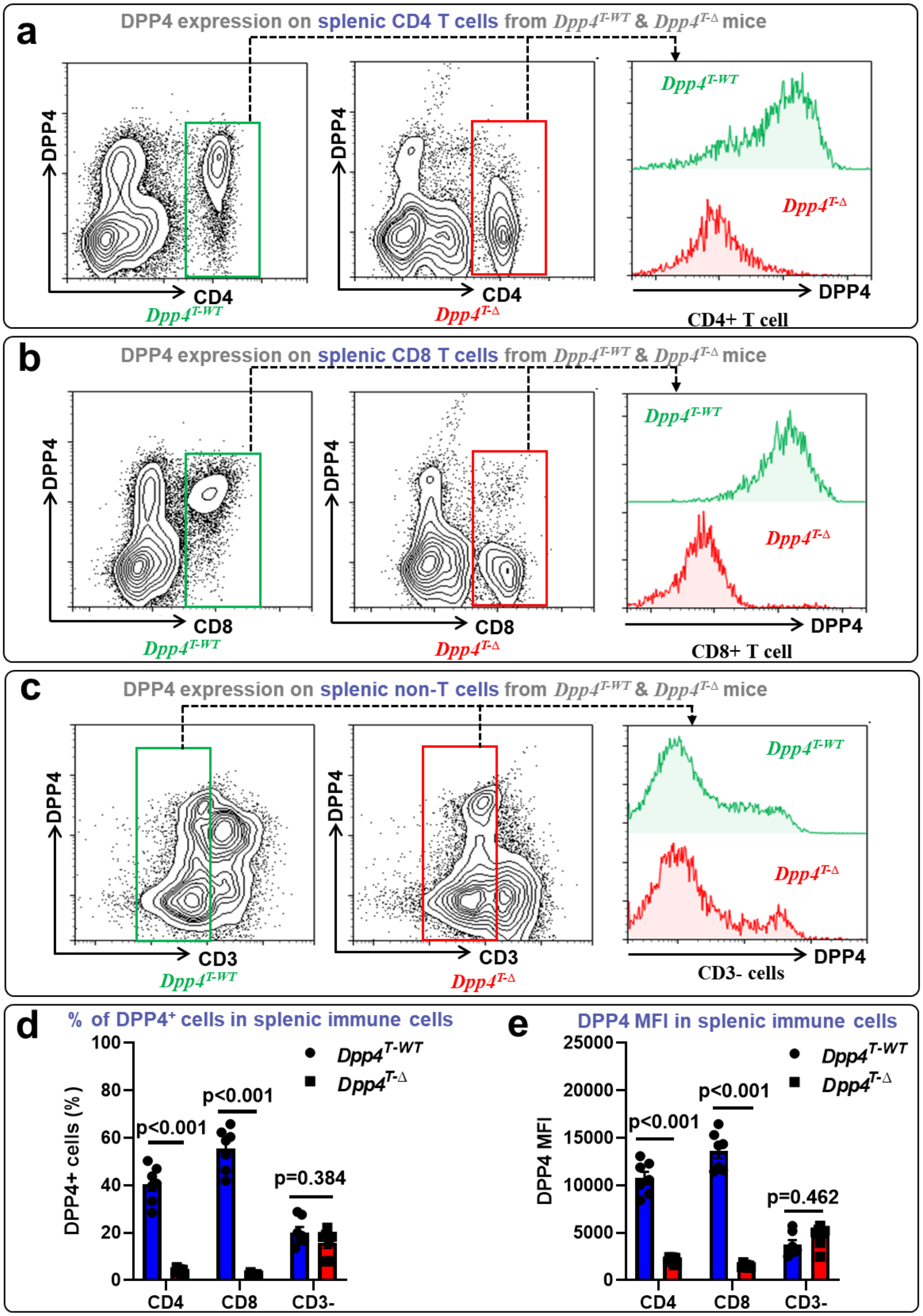

Fig. S8 DPP4 expression on splenocytes in *Rag1*$^{-/-}$ mice adoptively transferred with wild-type or DPP4 deficient T cell: *Rag1*$^{-/-}$ mice lacking lymphocytes were adoptively

transferred with T cells isolated from *Dpp4*$^{+/+}$ or *Dpp4*$^{-/-}$ mice and intravenously injected with proprotein convertase subtilisin/kexin type 9 (PCSK9)-overexpressing AAV8 virus, followed by 16 weeks of high fat diet feeding to induce atherosclerosis. Splenocytes were isolated for flow cytometric detection of DPP4 expression. **a** – **c**, Contour plots and histograms showing the expression of DPP4 on $CD4^+$ T cells (**a**), $CD8^+$ T cells (**b**), and $CD3^-$ cells (**c**). **d & e**, Percentages of $DPP4^+$ cells (**d**) and mean fluorescence intensity of DPP4 (**e**) in $CD4^+$ T cells, $CD8^+$ T cells, and $CD3^-$ cells. N=7 mice/group.

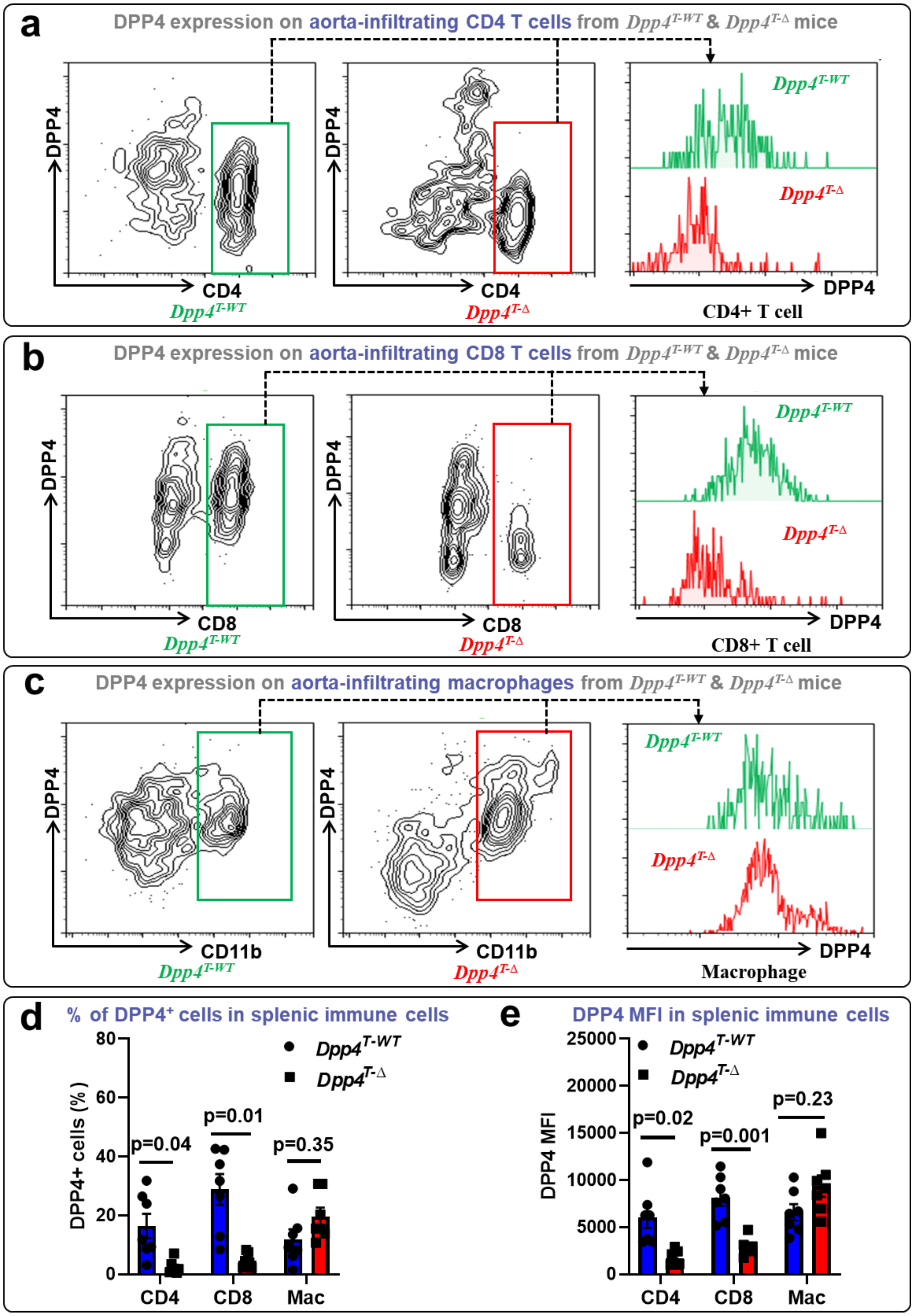
a
DPP4 expression on aorta-infiltrating CD4 T cells from Dpp4T-WT & Dpp4T-Δ mice
DPP4
CD4
Dpp4T-WT
Dpp4T-Δ
DPP4
CD4+ T cell
b
DPP4 expression on aorta-infiltrating CD8 T cells from Dpp4T-WT & Dpp4T-Δ mice
CD8
CD8+ T cell
c
DPP4 expression on aorta-infiltrating macrophages from Dpp4T-WT & Dpp4T-Δ mice
CD11b
Macrophage
d
% of DPP4+ cells in splenic immune cells
DPP4+ cells (%)
p=0.04
p=0.01
p=0.35
CD4
CD8
Mac
e
DPP4 MFI in splenic immune cells
DPP4 MFI
p=0.02
p=0.001
p=0.23

Fig. S9 DPP4 expression on aorta-infiltrating immune cells in *Rag1*$^{-/-}$ mice adoptively transferred with wild-type or DPP4 deficient T cell: *Rag1*$^{-/-}$ mice lacking lymphocytes were adoptively transferred with T cells isolated from *Dpp4*$^{+/+}$ or *Dpp4*$^{-/-}$ mice and intravenously injected with proprotein convertase subtilisin/kexin type 9 (PCSK9)-overexpressing AAV8 virus, followed by 16 weeks of high fat diet feeding to induce atherosclerosis. Aortic tissue was then isolated and digested using collagenase to prepare single cell suspension, followed by flow cytometric detection of DPP4 expression. **a – c**, Contour plots and histograms showing the expression of DPP4 on CD4$^+$ T cells (**a**), CD8$^+$ T cells (**b**), and CD11b$^+$ cells (**c**). **d & e**, Percentages of DPP4$^+$ cells (**d**) and mean fluorescence intensity of DPP4 (**e**) in CD4$^+$ T cells, CD8$^+$ T cells, and CD11b$^+$ cells (macrophage, Mac). N=7 mice/group.

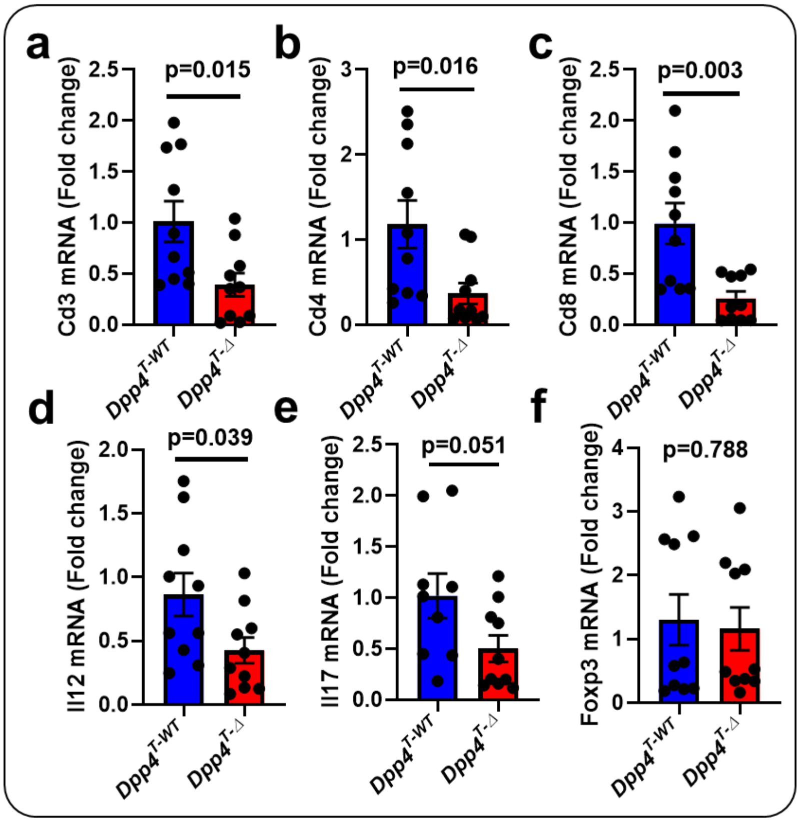

Fig. S10 Real-time PCR analysis of markers for T cell and its subsets in the aorta tissues of *Dpp4*$^{T\text{-}\Delta}$ and *Dpp4*$^{T\text{-}WT}$ mice: *Dpp4*$^{T\text{-}\Delta}$ and *Dpp4*$^{T\text{-}WT}$ mice infected with PCSK9-expressing AAV were fed a high fat diet for 16 weeks. Expressions of T cell marker genes *Cd3* (**a**), *Cd4* (**b**), *Cd8* (**c**), Th1 gene *Il12* (**d**), Th17 gene *Il17* (**e**), and Treg gene *Foxp3* (**f**) were detected by real-time PCR. The aortas used for the PCR analysis were from 2 batches of independent experiments. N=10/group.

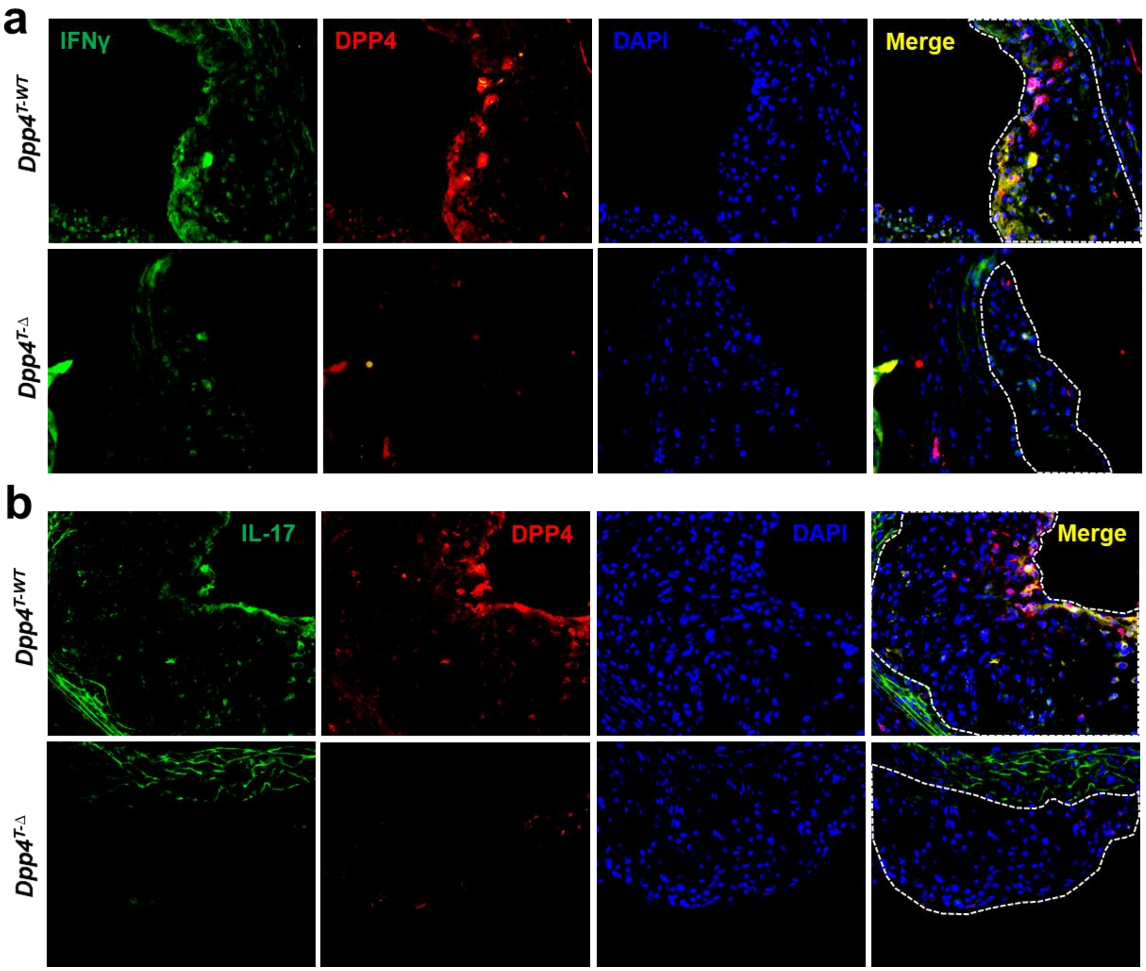

Fig. S11 IFNγ and IL-17 expression in aortic plaque lesions of *Dpp4*$^{T\text{-}\Delta}$ and *Dpp4*$^{T\text{-}WT}$ mice: *Dpp4*$^{T\text{-}\Delta}$ and *Dpp4*$^{T\text{-}WT}$ mice infected with PCSK9-expressing AAV were fed a high fat diet for 16 weeks. The aortic sinus sections were stained with anti-mouse DPP4 (red) and anti-mouse IFNγ (green, **a**) or IL-17 (green, **b**) and visualized under a fluorescence microscope (400x magnification).

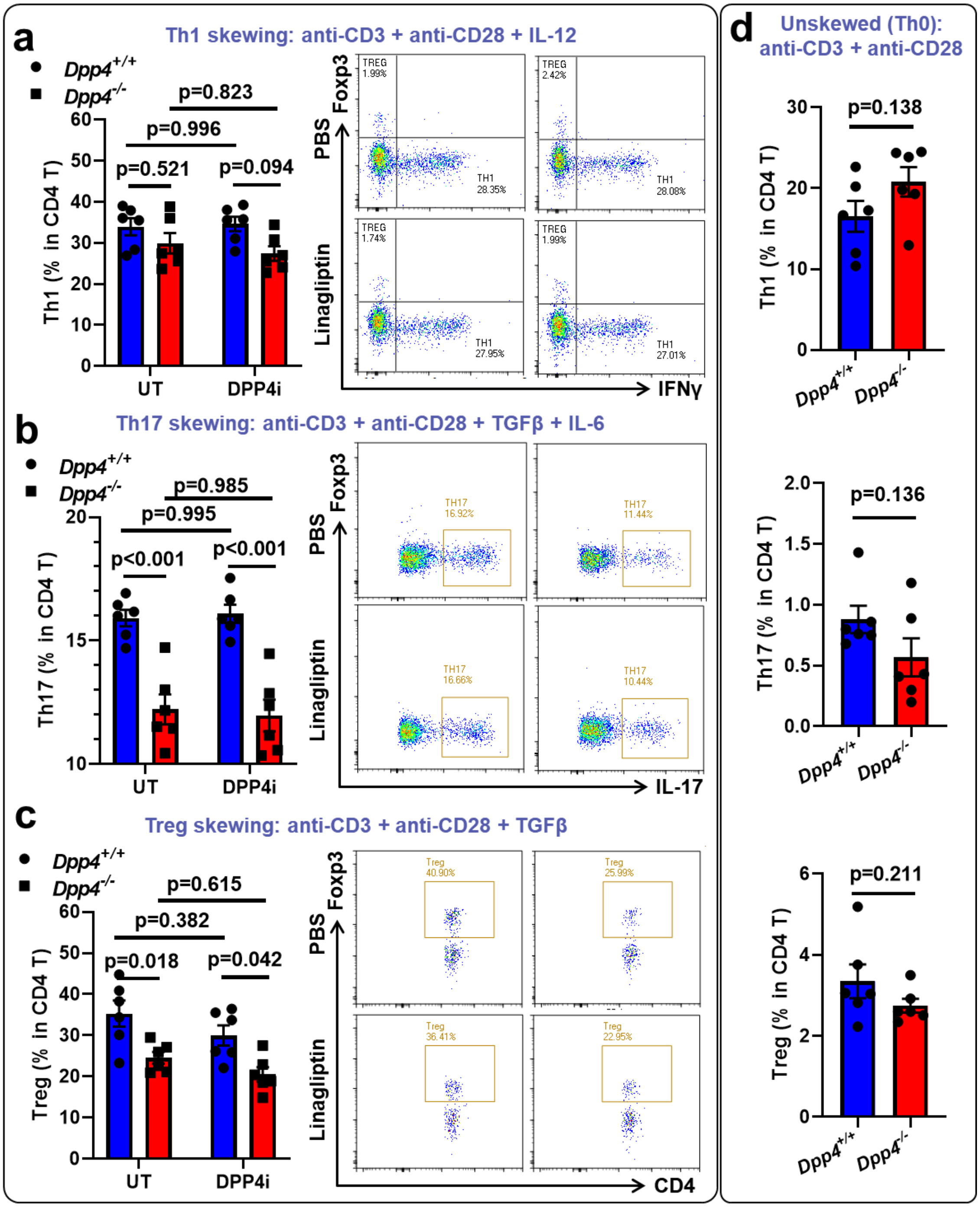

Fig. S12 Effect of DPP4 deficiency on Th1, Th17, and Treg differentiation: T cells isolated from *Foxp3-GFP* (*Dpp4*$^{+/+}$) and *Foxp3-GFP Dpp4*$^{-/-}$ (*Dpp4*$^{-/-}$) mice were treated with 1 µg/mL anti-CD3 antibody, 1 µg/mL anti-CD28 antibody, under the skewing conditions of Th1 (10 ng/mL IL-12, **a**), Th17 (5 ng/mL TGFβ plus 20 ng/mL IL-6, **b**), Treg (5 ng/mL TGFβ, **c**), or Th0 (without skewing cytokines, **d**) for 3 days. DPP4 enzymatic inhibitor linagliptin or vehicle control (PBS) was also supplemented into the culture system to delineate the enzymatic and non-enzymatic effects. The frequencies of Th1, Th17, and Treg were examined using flow cytometry. N=6/group.

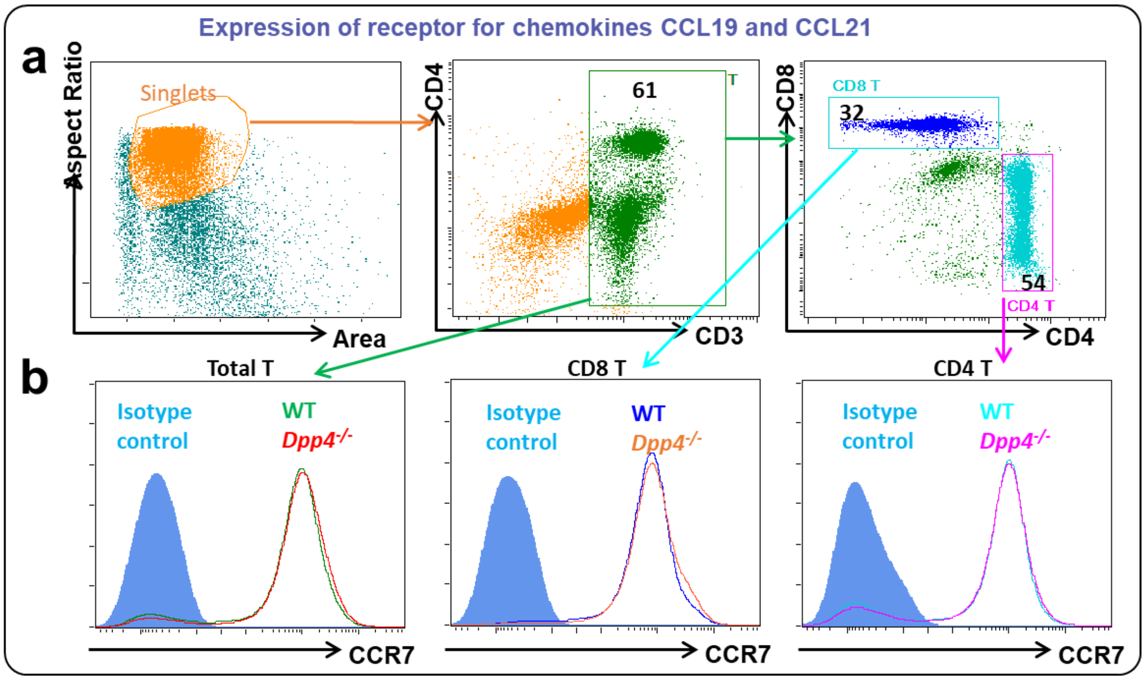

Fig. S13 Expression of chemokine receptor CCR7 on *Dpp4*$^{+/+}$ and *Dpp4*$^{-/-}$ T cells: *Dpp4*$^{+/+}$ and *Dpp4*$^{-/-}$ lymph node cells were stained with CCR7 and markers for T cell subsets. Flow cytometry showed that DPP4 deficiency did not affect the expression of CCR7, the chemokine receptor for CCL19 and CCL21, suggesting that the reduced migratory activities towards CCL19 and CCL21 in *Dpp4*$^{-/-}$ mice was independent of chemokine receptor expression. **a**, Gating strategy; **b**, Expression of CCR7 on total T cells, CD8^{+} T cells and CD4^{+} T cells.

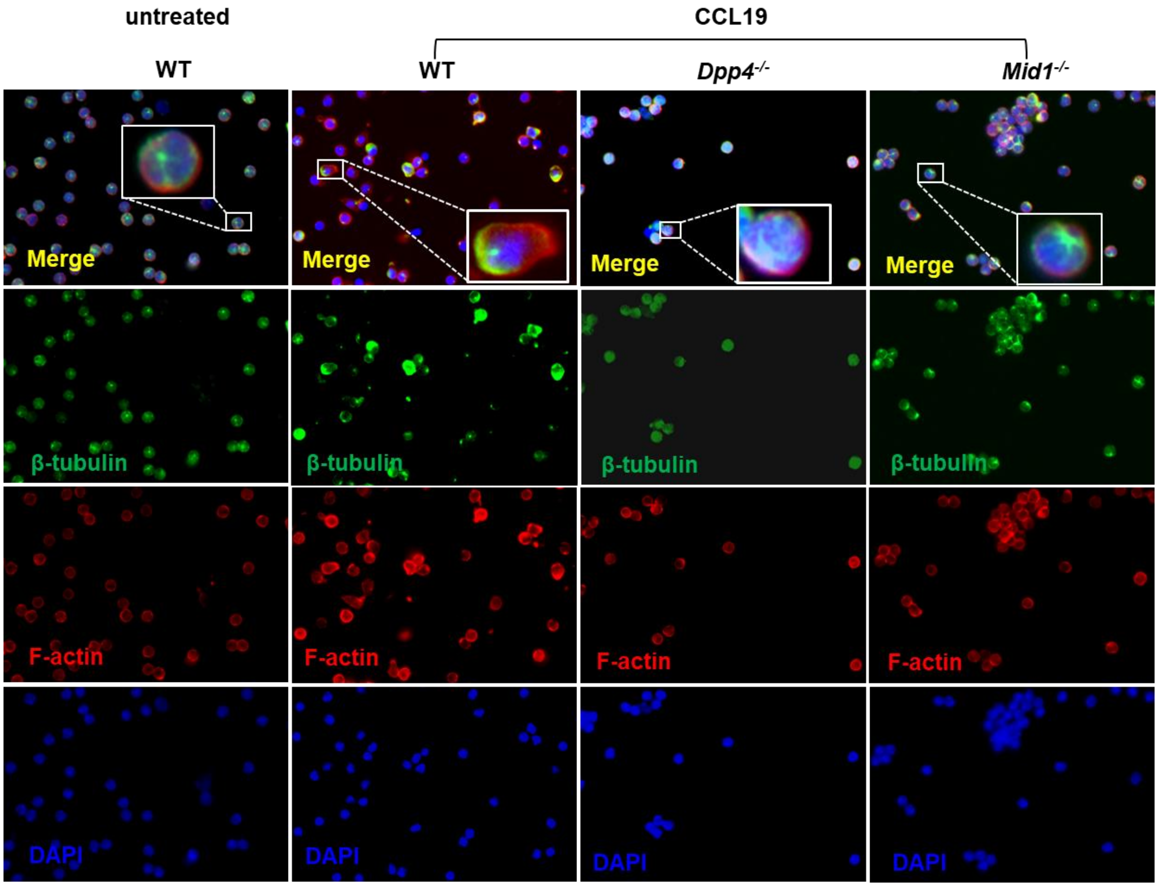

Fig. S14 Chemokine-induced morphological change and polarization in *Dpp4*$^{-/-}$ and *Mid1*$^{-/-}$ T cells: WT, *Dpp4*$^{-/-}$, and *Mid1*$^{-/-}$ T cells were isolated from lymph node and incubated on 2μg/mL mouse ICAM-1 pre-coated slides for 20 min. After gentle washes with 1x PBS, adhered T cells were treated with 0.5 μg/mL CCL19 or PBS for 10 min, followed by staining of F-actin (red) and β-tubulin (green). Representative images showed that CCL19 induced cell shape change and polarization of F-actin/β-tubulin in WT T cells, which were largely suppressed in *Dpp4*$^{-/-}$ and *Mid1*$^{-/-}$ T cells.

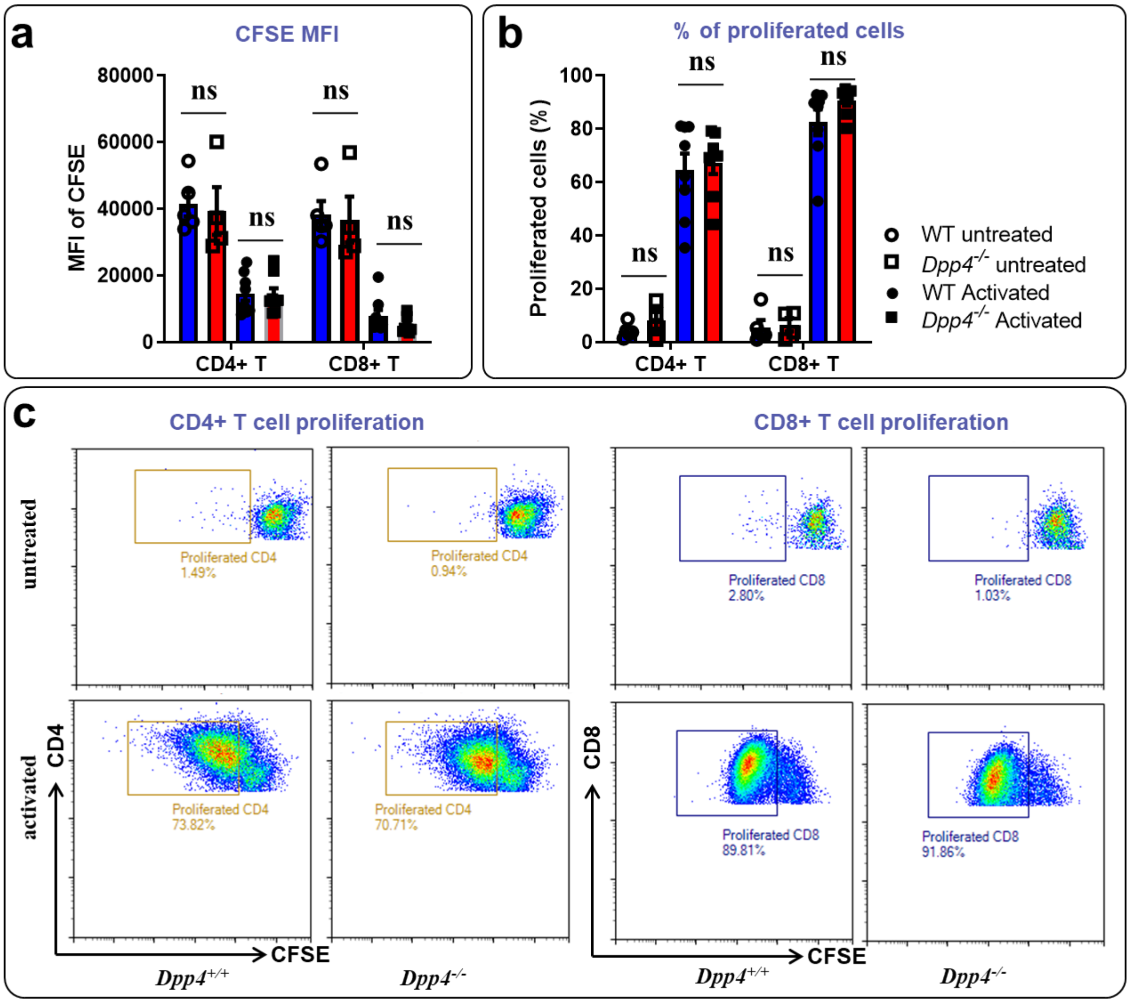

Fig. S15 DPP4 deficiency does not affect T cell proliferation: T cells isolated from *Dpp4*$^{+/+}$ and *Dpp4*$^{-/-}$ mice were labeled with 1 μM CFSE and then either untreated or stimulated with anti-CD3 and anti-CD28 antibodies. Cells were collected and stained with PERCP-labeled anti-CD4, and PE-labeled anti-CD8 antibodies 72 hours after stimulation. Proliferation was detected by flow cytometry. Mean fluorescent intensity (MFI) of CFSE and percentages of proliferated CD4^{+} and CD8^{+} T cells were analyzed. **a**, CFSE MFI of untreated or activated *Dpp4*$^{+/+}$ and *Dpp4*$^{-/-}$ T cells. **b**, Percentages of proliferated *Dpp4*$^{+/+}$ and *Dpp4*$^{-/-}$ T cells. **c**, Representative dot plots showing the intensity of CFSE in CD4^{+} or CD8^{+} T cells from *Dpp4*$^{+/+}$ or *Dpp4*$^{-/-}$ mice. ns, not significant.

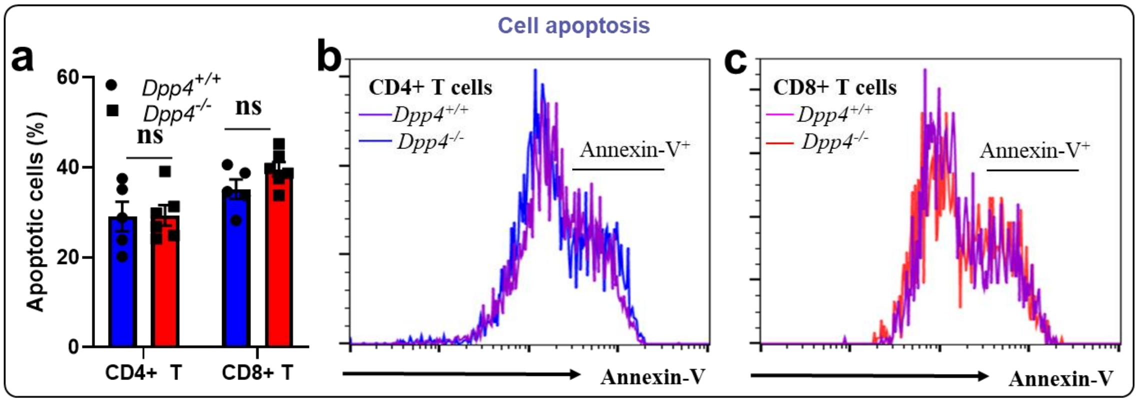

Fig. S16 DPP4 deficiency does not affect T cell apoptosis: T cells isolated from *Dpp4*$^{+/+}$ and *Dpp4*$^{-/-}$ mice were stimulated with anti-CD3 and anti-CD28 antibodies. Cells were stained with FITC-labeled annexin-V, PERCP-labeled anti-CD4, and APC-labeled anti-CD8 antibodies after 5 days of culture. Apoptosis was detected by flow cytometry. Statistical analysis of apoptotic cells (**a**) and representative histogram (**b**, CD4+ T cells; **c**, CD8+ T cells) were shown. ns, not significant.

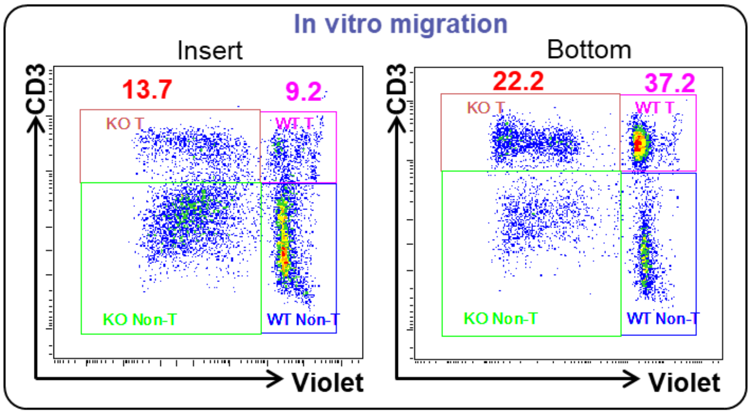

Fig. S17 Flow cytometric detection of *Mid1*$^{+/+}$ or *Mid1*$^{-/-}$ T cells in the insert and bottom well of Transwell® assay: Splenic T cells isolated from *Mid1*$^{+/+}$ or *Mid1*$^{-/-}$ mice were labeled with CellTrace™ Violet and CFSE respectively. Cells were then mixed at 1:1 ratio and placed in the insert of a Transwell® plate with CCL19 in the bottom well. Cells migrated to the bottom well and those remaining in the insert were collected for flow cytometric detection. Representative dot plots showing percentages of *Mid1*$^{+/+}$ and *Mid1*$^{-/-}$ T cells migrated to the bottom well and those remained in the insert of the Transwell® plate.

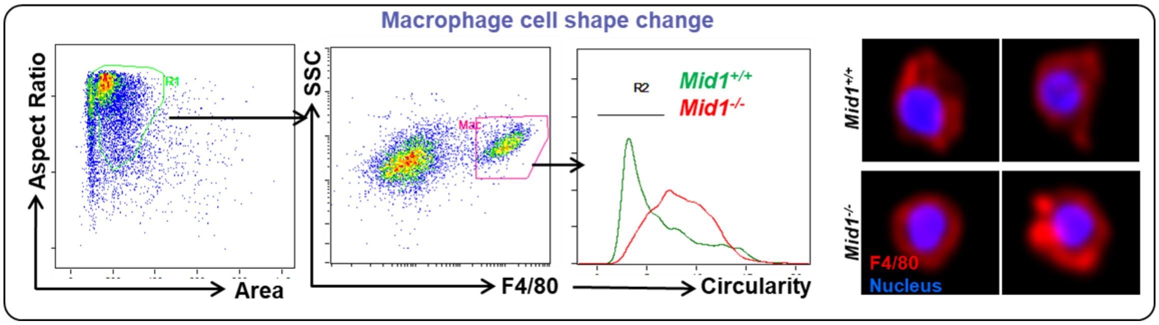

Fig. S18 Morphological changes of *Mid1*$^{+/+}$ and *Mid1*$^{-/-}$ macrophages upon chemokine stimulation: Morphological changes of *Mid1*$^{+/+}$ and *Mid1*$^{-/-}$ peritoneal macrophages in response to 30-min complement component 5α (C5α) treatment were detected by imaging flow cytometry. Left, dot plots and histogram showing the circularity of macrophages after C5α stimulation; Right, representative images.
